# Benzoylselenoureas: A Novel Dual-Action Inhibitor Targeting Fungal Growth and Urease Activity in *Cryptococcus neoformans*

**DOI:** 10.1021/acsomega.5c06398

**Published:** 2025-09-15

**Authors:** Nathália Evelyn Morais Costa, Thayná Lopes Barreto, Nathalia Monteiro Lins Freire, Júlio Cosme Santos da Silva, Thiago Mendonça de Aquino, Eduardo E. Alberto, Kelly Ishida, Ângelo de Fátima

**Affiliations:** † Department of Chemistry, Institute of Exact Sciences, 28114 Universidade Federal de Minas Gerais, Belo Horizonte 31270-901, MG, Brazil; ‡ Department of Microbiology, Institute of Biomedical Sciences, Universidade de São Paulo, Sao Paulo 05508-090, SP, Brazil; § Research Group on Therapeutic Strategies—GPET, Institute of Chemistry and Biotechnology, 28112 Universidade Federal de Alagoas, Maceio 57072-970, AL, Brazil; ∥ Institute of Chemistry and Biotechnology, Universidade Federal de Alagoas, Maceio 57072-900, AL, Brazil

## Abstract

*Cryptococcus neoformans* cause cryptococcal meningitis, particularly in individuals with compromised immune systems. In this context, urease plays a crucial role in fungal survival by facilitating infection spread and penetration of the blood–brain barrier, making this enzyme a potential target for antifungal therapy. Eleven benzoylselenoureas (**BSU**) were synthesized in 15–75% yields via a one-pot approach using benzoyl chloride, KSeCN, and anilines containing electron-donating or electron-withdrawing groups. The antifungal and urease inhibitory activities of these compounds were evaluated against *C. neoformans*. For comparison, the corresponding benzoylthioureas (**BTU**) analogs were also synthesized to assess the influence of the chalcogen atom on biological activity. Antifungal activity was determined using the broth microdilution assay, while urease inhibition was evaluated through ammonia quantification. Additionally, inhibitor-enzyme interactions were investigated using homology modeling, molecular docking, molecular dynamics simulations, and density functional theory (DFT) calculations. The **BSU** compounds demonstrated higher antifungal activity than their **BTU** analogs, with minimum inhibitory concentration (MIC) and minimum fungicidal concentration (MFC) values ranging from 1 to 16 mg/L (except for **BSU3**, **BSU8**, and **BSU11**). The most active compounds against yeast were **BSU1** and **BSU5**, which feature an OMe group at the *meta* position of the aniline moiety. Furthermore, **BSU** compounds exhibited strong urease inhibition, with ureIC_50_ values ranging from 0.95 to 13.95 nM. Computational studies revealed that **BSU** compounds predominantly coordinate with the Ni­(II) center in a bidentate mode, likely involving the amide oxygen and selenium atoms. These findings indicate that **BSU** compounds effectively inhibit urease activity and *C. neoformans* growth at low concentrations, reinforcing urease as a promising target for antifungal therapy. Molecular modeling confirmed the strong affinity of **BSU** compounds for urease, supporting their potential as novel antifungal agents.

## Graphical abstract

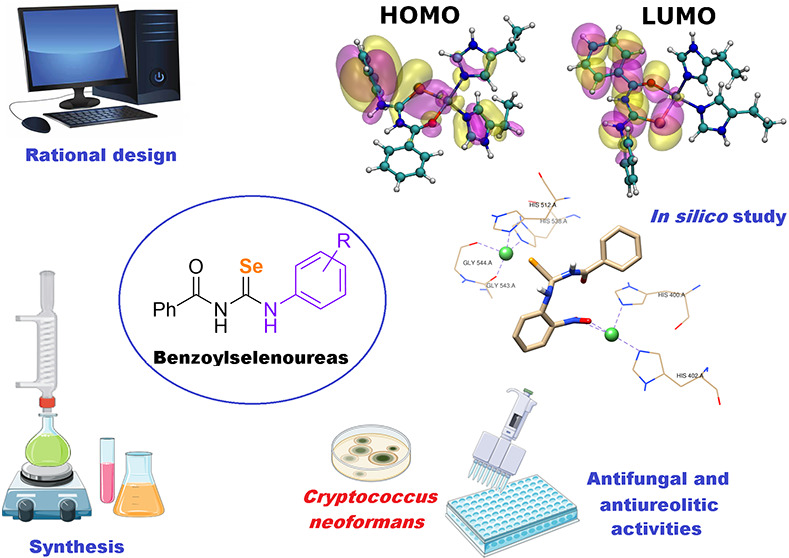

## 1 Introduction

*Cryptococcus neoformans* is a fungal pathogen that causes cryptococcal meningitis (CM), a life-threatening fungal infection.^1^ Recently classified as a critical priority pathogen by the World Health Organization (WHO),^2^ it poses a significant threat to immunocompromised individuals, particularly those with HIV. Global estimates indicate approximately 152,000 annual cases with a staggering mortality rate exceeding 74%.^3^ Infection typically begins with inhalation of environmental spores or yeast cells, leading to pulmonary infection. Although the initial infection may appear localized, *C. neoformans* can disseminate throughout the body, with a particular tropism for the central nervous system (CNS).^1^ This systemic spread is facilitated by multiple fungal virulence factors, including: (i) cell wall components; (ii) the protective polysaccharide capsule; (iii) melanin production; (iv) enzymatic activity (such as urease activity); (v) adhesion capability and (vi) biofilm formation.^4^ These virulence mechanisms collectively promote fungal dissemination and subsequent CNS invasion.

Currently, the World Health Organization (WHO) recommends treating cryptococcosis with a combination of three drugs—amphotericin B, fluconazole, and flucytosine—administered either individually or in combination. However, despite these therapeutic options, pharmacological choices remain limited, and increasing resistance of *Cryptococcus* to fluconazole has been reported. Given the high incidence and mortality rates associated with cryptococcosis, there is an urgent need for novel antifungal therapies.^5^ The establishment and spread of *C. neoformans* infection in the central nervous system (CNS) are facilitated by virulence factors that enhance fungal survival under host conditions.^6^ A key factor is urease, an enzyme that catalyzes the hydrolysis of urea into ammonia. Urease plays a critical role in cryptococcal meningitis (CM) pathogenesis by weakening the blood–brain barrier (BBB) and promoting CNS invasion.^7,8^ Since urease is absent in humans, its inhibition presents an attractive antifungal target and a promising therapeutic strategy.^4^

Chalcogen derivatives have demonstrated significant antifungal and urease inhibition activity. In 2019, Braga *et al.* showed that Biginelli adducts derived from thioureas (**1**; Figure 1) exhibited stronger *Canavalia ensiformis* urease inhibition than those derived from urea.^9^ Earlier, in 2015, Brito *et al.* reported that benzoylthioureas (**BTUs**) also displayed urease inhibition activity in *C. ensiformis* (Figure 1).^10^ Angeli and collaborators investigated the antifungal activity of various selenourea derivatives (**2**; Figure 1).^11^ Their results revealed that selenium-containing organic frameworks exhibited antifungal activity comparable to standard drugs. However, this activity was suppressed when selenium was replaced by its isosteric elements, sulfur or oxygen. Some compounds tested by Angeli and collaborators showed excellent selectivity against *Malassezia pachydermatis* compared to *Malassezia furfur* and *Malassezia globosa*. Moreover, at the tested concentrations, these derivatives did not exhibit cytotoxicity against healthy human cells, highlighting their potential as promising new antifungal agents.^11^ De Jesus and collaborators further demonstrated the fungicidal potential of selenium-containing organic compound (**3**; Figure 1) through studies involving a quinoline derivative.^12^ The compound **3** inhibited key virulence factors of *C. neoformans*, including fungal melanization by suppressing laccase activity. Additionally, it hindered dispersed biofilm cells and polysaccharide capsule formation.^12^

**Figure 1:**
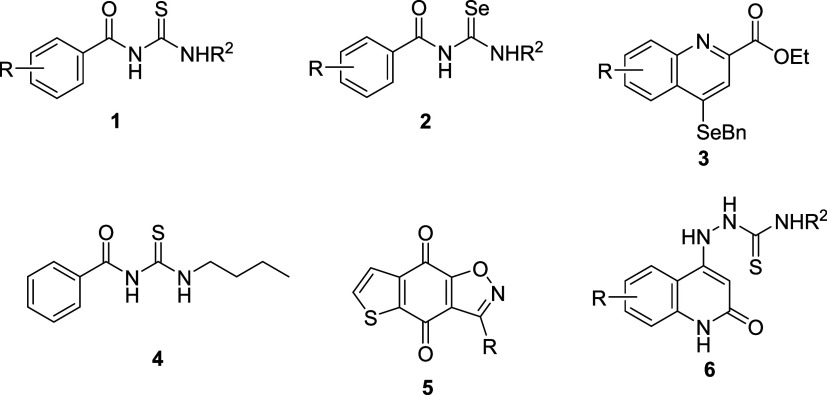
Organocalcogen compounds that exhibited urease inhibition activity and/or antifungal activity.

Although studies on fungal urease inhibition remain limited, recent reports highlight promising antifungal activity against *C. neoformans*.^13^ Compound **4** (Figure 1) demonstrated an MIC of 31.25–62.5 mg/L and exhibited synergistic effects when combined with amphotericin B.^13^ In another study, Li *et al.* identified a series of tricyclic isoxazole derivative (**5**; Figure 1) that showed anticryptococcal activity, inhibiting *C. neoformans* growth and displaying fungicidal effects. Notably, this compound also targeted key virulence factors, including biofilm formation, melanin production, and urease activity.^14^ Furthermore, Elbastawesy *et al.* reported that quinolin-2-one thiosemicarbazide derivatives (**6**; Figure) effectively inhibited *Rhodotorula mucilaginosa*, a urease-producing basidiomycete fungus.^15^ These findings collectively support urease as a viable target for developing novel antifungal therapies.

In this study, we investigated whether organoselenium compounds demonstrate enhanced *Cryptococcus*-urease inhibitory activity compared to their organosulfur analogs. To address this question, we designed and synthesized a series of benzoylselenoureas (**BSU**) and assessed their biological activity against both *C. neoformans* yeasts and urease enzyme. The antifungal efficacy of these selenium-containing compounds was systematically compared with that of their corresponding benzoylthioureas (**BTU**). The synthesis protocol for the **BTU** analogs employed in this comparative study has been previously reported.^10^

## 2 Results and Discussion

### 2.1 Synthesis of Benzoylselenoureas and Benzoylthioureas

Considering previous reports on the biological activity of organochalcogen compounds, we designed a series of benzoylselenourea derivatives (**BSU1-BSU11**) featuring electron-donating (EDG) or electron-withdrawing groups (EWG) on the aromatic ring of the aniline moiety to further explore their biological properties. The synthesis of **BSU1-BSU11** involved the addition of KSeCN to benzoyl chloride (**7**), generating the isoselenocyanate intermediate **8**, which was subsequently trapped by aniline derivatives (**9**) (Scheme 1). No clear correlation was observed between the electronic nature of the aniline substituents and the product yields. Benzoylselenoureas bearing either EDGs (**BSU1**-**BSU5**) or EWGs (**BSU6**- **BSU11**) were obtained in 15–67% yields (Table 1). Except for **BSU-8**, all other compounds were previously unreported.^16^ To investigate the influence of the chalcogen atom on biological activity, we also synthesized the corresponding benzoylthiourea analogues **BTU1**-**BTU11** following a previously reported method.^10^

**Scheme 1:**
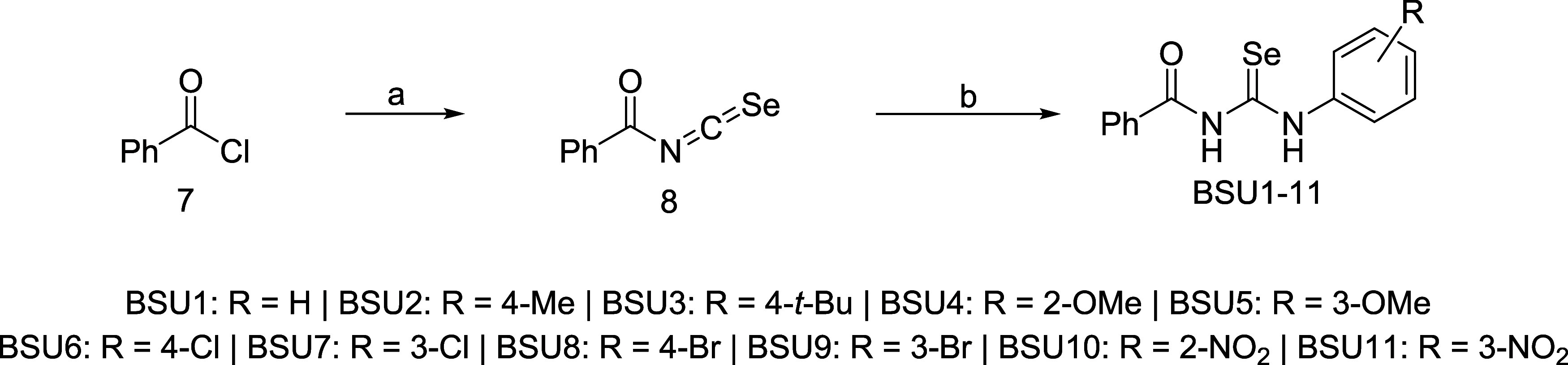
Synthetic Approach for Obtaining the Benzoylselenoureas of **BSU1-BSU11^a^**

a Reagents and reactions conditions: (a) KeSeCN, 50 °C, 30 min; (b) ArNH_2_, acetone.

**Table 1:**
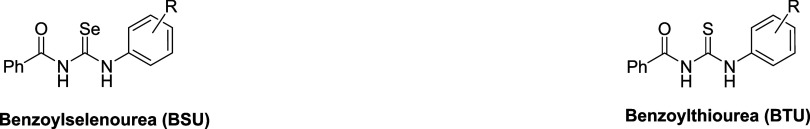
Biological Assessments of the Benzoylselenoureas (**BSU**) and Benzoylthioureas (**BTU**) in *C. neoformans*

			MIC (mg/L)^a^					MIC (mg/L)^a^			
	R	yield (%)	RPMI	urea	MIC_ure_ ^b^ (mg/L)	MFC^c^ (mg/L)	ureIC_50_ ^d^ (nM/μg/L)	RPMI	urea	MIC_ure_ ^b^ (mg/L)	MFC^c^ (mg/L)
**1**	H	75	2	0.06	0.06	2	4.88/1.48	>128	32	32	>128
**2**	4-Me	15	16	0.5	0.5	16	1.07/0.34	>128	32	<0.25	>128
**3**	4-*t-*Bu	35	>128	>128	>128	>128	0.95/0.34	8	1	0.5	>128
**4**	2-OCH_3_	67	8	0.5	0.5	8	2.33/0.78	>128	64	32	>128
**5**	3-OCH_3_	25	1	0.12	0.12	1	13.95/4.65	32	4	4	>128
**6**	4-Cl	66	16	0.5	0.25	16	3.02/1.02	>128	1	<0.25	>128
**7**	3-Cl	56	8	0.25	0.12	16	7.59/2.56	>128	4	<0.25	>128
**8**	4-Br	40	128	8	8	>128	4.04/1.54	>128	32	<0.25	>128
**9**	3-Br	30	4	0.12	0.06	4	2.31/0.88	>128	32	2	>128
**10**	2-NO_2_	23	8	1	1	16	8.68/3.02	>128	>128	>128	>128
**11**	3-NO_2_	37	32	1	1	64	7.91/2.76	>128	>128	>128	>128

a Minimum inhibitory concentration (MIC) required to inhibit 50% of fungal growth in RPMI or urea media.

b Minimum inhibitory concentration (MIC_ure_) required to inhibit 100% yeast urease activity in urea medium.

c Minimum fungicidal concentration (MFC) values obtained after assay in RPMI medium.

d Inhibitory concentration of 50% (ureIC_50_) for the urease activity in protein crude extract.

### 2.2 Inhibitory Effect of Benzoylselenoureas BSU1–11 on Fungal Growth and Urease Activity of *C. neoformans*

The biological activity of **BSU1-BSU11** and its **BTU1-BSU11** analogues was evaluated against *C. neoformans* growth and urease activity (Table 1). **BSU1-BSU11** inhibited *C. neoformans* growth with minimum inhibitory concentration (MIC) values ranging from 1 to >128 mg/L in RPMI standard medium. Among the tested compounds, **BSU1** and **BSU5** exhibited the strongest inhibitory and fungicidal effects, while **BSU3** showed the weakest activity against yeast growth (Table 1, entries 1, 5, and 3, respectively). Screening of **BTU1-BTU11** under identical conditions revealed significantly lower activity compared to the **BSU** analogues, with MIC values ranging from 8 to >128 mg/L. Notably, all benzoylselenourea derivatives demonstrated fungicidal activity (with minimum fungicidal concentration, MFC, values of 1–64 mg/L), except for **BSU3** and **BSU8**.

The inhibitory activity against yeast urease was assessed using Christensen’s medium, which contains urea to induce *URE1* gene expression.^17^ This medium served to complement fungal growth inhibition data by specifically demonstrating urease activity inhibition in yeast cells. Even in the presence of fungal growth, inhibition of urease activity was evidenced by the absence of medium color change, indicating suppressed ammonia production. All benzoylselenourea derivatives except **BSU3** and **BSU8** showed potent urease inhibition, with minimum inhibitory concentration (MIC_ure_) values ranging from 0.06 to 1 mg/L against yeast urease. In contrast, benzoylthiourea derivatives (**BTU1**-**BTU9**) exhibited weaker activity, showing fungal growth inhibition at MICs of 1–64 mg/L and urease inhibition at MIC_ure_ values of <0.25 to 32 mg/L (Table 1). These results demonstrate the superior potency of benzoylselenoureas (**BSU**) in inhibiting intracellular urease activity in *C. neoformans* compared to their benzoylthiourea counterparts.

The unsubstituted **BSU1** derivative demonstrated the strongest activity against both fungal growth and urease activity in *C. neoformans*, along with fungicidal effects. Among the substituted derivatives, we observed that 3-position modifications generally produced more effective analogs than 4-position substitutions, with **BSU5** showing particularly notable activity (Table 1). Importantly, we found a significant positive correlation (Pearson’s *r* = 0.70, *p* = 0.016) between MIC values (fungal growth inhibition) and MIC_ure_ values (urease inhibition) for the **BSU** compounds against *C. neoformans*, suggesting a relationship between these two inhibitory activities.

Given the promising antifungal activity of benzoylselenoureas (**BSU1**-**BSU11**), we evaluated their ability to inhibit urease activity using a crude protein extract from *C. neoformans*. The compounds exhibited potent inhibition, with ureIC_50_ values ranging from 0.95 to 13.95 nM—significantly lower than previously reported for *N*-(butylcarbamothioyl)­benzamide (ureIC_50_ = 53.3 mg/L against *C. neoformans* urease)^13^ and their benzoylthiourea (**BTU**) analogs (IC_50_ ∼ 0.5 mM against *C. ensiformis* urease).^10^ These results highlight the superior inhibitory potency of selenium-containing compounds against both fungal cellular urease and the purified enzyme, reinforcing their potential as targeted antifungals.

While all tested benzoylselenoureas (**BSU**) demonstrated potent inhibition of *C. neoformans* urease in enzymatic assays, certain derivatives showed diminished activity against either fungal growth or intracellular urease. This observed discrepancy likely stems from variations in the compounds’ chemical properties that affect their cellular penetration through the fungal capsule, cell wall, and plasma membrane. A notable example is **BSU3**, which contains a 4-butyl substituent—while it exhibited the lowest ureIC_50_ value (0.95 nM) against urease in fungal lysate (Table 1), its bulky substituent may impair cellular uptake, resulting in reduced biological activity against intact fungal cells.

Previous studies have identified few compounds capable of simultaneously inhibiting both fungal growth and urease activity.^13,14,18^ Although organoselenium compounds have demonstrated significant antifungal activity against pathogenic fungi including *Cryptococcus* species,^11,12,19^ their potential to inhibit urease activity has remained unexplored. Our study now reveals for the first time that benzoylselenourea (**BSU**) derivatives exhibit this dual inhibitory capacity, showing potent activity against both *C. neoformans* growth (at micromolar concentrations) and urease activity (at nanomolar).

### 2.3 Homology Modeling, Molecular Docking, and Dynamics Simulations, and DFT Calculations

Based on the alignment procedure, the homologous model for *C. neoformans* urease was created while preserving the coordinate mapping between the target and the template in the monomeric oligostate mode. When superimposing the Jack Bean urease structure with the homology model, the RMSD value was found to be 0.365. The global and per-residue model quality was assessed using the QMEAN scoring function. The Global Model Quality Estimate (GMQE) was 0.84, while the QMEANDisCo global score was 0.80 ± 0.05. The Ramachandran plot analysis revealed that 93.28% of all residues were located within 98% of the most favorable regions. Figure 2A illustrates the residues that exhibited the highest number of interactions with the studied derivatives and the Ni metals in the active site of the predicted target.

**Figure 2:**
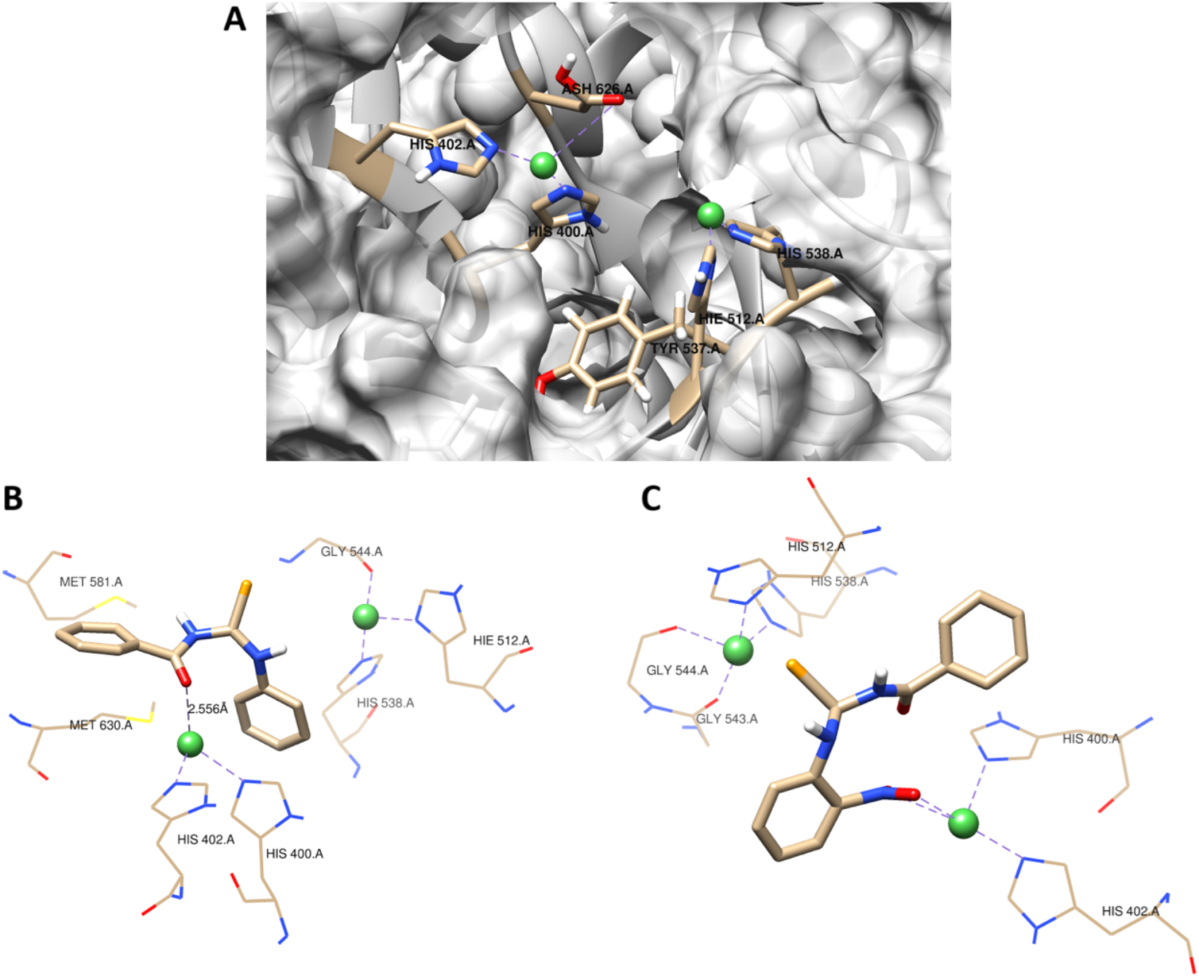
(A) Schematic representation of the active site of *C. neoformans* urease obtained through homology modeling. 3D representation of interactions between **BSU1** (B) and **BSU10** (C) with *C. neoformans* urease. The binding conformation of the ligands is visualized using a stick representation, while green spheres denote the two Ni metals. The coordination with metal, hydrophobic, and ionic interactions is illustrated using dotted lines.

The MD simulations involving **BSU1-BSU11** revealed two pivotal coordination mechanisms at the urease active site across all derivatives. The first mechanism involves the direct coordination of the carbonyl group in **BSU1-BSU9** and **BSU11** with the Ni­(II) centers via its oxygen atom, as illustrated in Figure 2B, which depicts the 3D interactions of **BSU1**. In contrast, the second mechanism is exclusive to the **BSU10** derivative, wherein coordination occurs through the oxygen atom of the nitro group (Figure 2C). These distinct coordination modes suggest two alternative pathways for interaction, which hold significant implications for specific biochemical processes and structural dynamics.

Moreover, throughout the simulation period, all complexes were stabilized by interactions with His400, with 64% of these interactions being ionic, and 18% each classified as hydrophobic and hydrogen bonding interactions. Additionally, 81% of the complexes were stabilized by hydrophobic contacts with Tyr537, while 72% of the derivatives interacted with His402, predominantly through ionic interactions, which accounted for 87% of the complexes. Figure 3A,B illustrate protein interactions with the representative compounds **BSU1** and **BSU10**, respectively. These findings highlight the critical role of these residues in ligand stabilization and underscore the relevance of specific interactions in maintaining complex stability.

**Figure 3:**
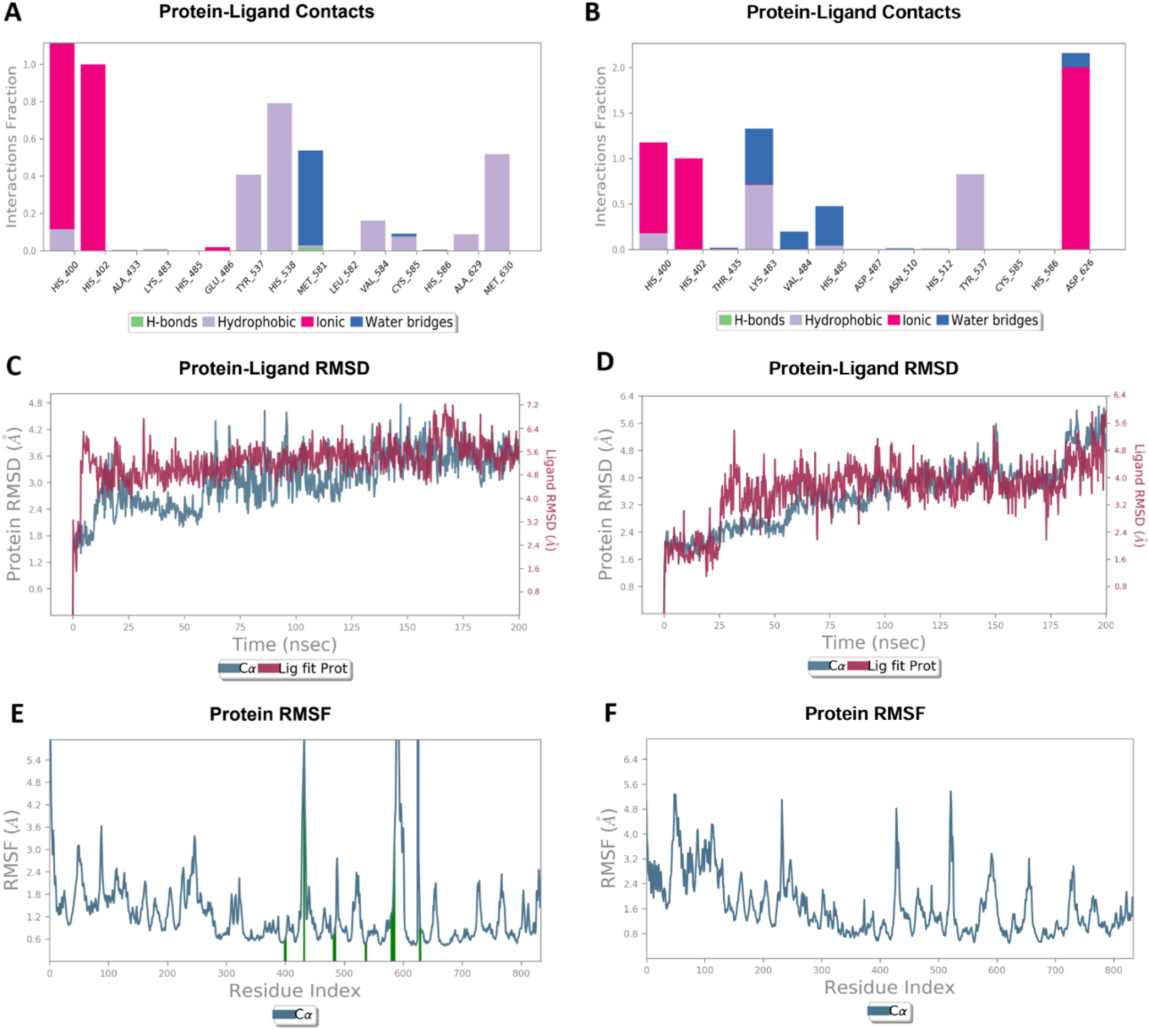
Protein interactions with the inhibitors **BSU1** (A) and **BSU10** (B) were monitored throughout the MD simulation. A value of 1.0 suggests that the specific interaction is sustained throughout 100% of the simulation, while values over 1.0 arise when the residue establishes numerous contacts of the identical subtype with the ligand. RMSD plots of the urease backbone and ligands **BSU1** (C) and **BSU10** (D) within the catalytic cavity of *C. neoformans* urease. RMSF plots of the urease complexed **BSU1** (E) and **BSU10** (F) in the catalytic site, where protein residues interacting with the ligand are indicated by green vertical bars.

The stability of the protein–ligand complexes was evaluated through Root Mean Square Deviation (RMSD) analysis, providing insights into structural fluctuations over 200 ns molecular dynamics (MD) simulations. Most complexes exhibited an initial adjustment phase within the first 20–30 ns, followed by stabilization. These results demonstrated that all ligands exhibited strong interactions, particularly those forming coordination bonds with Ni­(II) ions, which showed lower RMSD values, suggesting higher structural rigidity. Conversely, compounds with increased flexibility displayed slightly higher RMSD fluctuations but remained within an acceptable stability range. The Root Mean Square Fluctuation (RMSF) values were analyzed to assess the flexibility of individual residues within the urease-ligand complexes. As expected, higher fluctuations were observed in loop regions, while catalytic residues such as His400, His402, and Tyr537 remained relatively stable across all simulations. This outcome was expected, as these three residues are present in most interactions involving all complexes, indicating that ligand binding did not induce significant destabilization of the enzyme’s active site, reinforcing the structural compatibility of the tested inhibitors. RMSD and RMSF for **BSU1** and **BSU10** are shown in Figure 3C–F.

According to Pearson’s HSAB theory, as a soft base, selenium tends to form stable complexes with the Ni­(II) ion,^20^ which exhibits borderline acidity, further supporting its potential role in effective urease inhibition. To investigate these interactions further at the electronic level, Density Functional Theory (DFT) calculations were performed on the structures obtained from molecular dynamics simulations, incorporating and evaluating the explicit effects of electronic structure on the formation and stability of the investigated complexes. These calculations provide a deeper understanding of the electronic properties of selenium-containing inhibitors and their coordination modes in their interaction with the urease enzyme active site.

The representative structures displayed in Figure S1 (Supporting data) correspond to four selected complexes (**BSU1**, **BSU2**, **BSU7**, and **BSU10**) from the 11 investigated compounds. These were chosen to highlight the study’s key structural and electronic features. The HOMO and LUMO compositions for each complex are illustrated, shedding light on their electronic delocalization and potential reactivity.

From the DFT calculations, it was observed that the coordination mode of the complexes **BSU1** to **BSU9** follows a bidentate coordination pattern involving the amide oxygen and selenium atoms and nickel­(II) ions. This coordination motif is likely to contribute to the strong binding affinity and stability of these complexes with urease, as the electronic density distribution suggests an effective orbital overlap between the ligand and the metal center of the enzyme.

In contrast, the coordination mode of **BSU10** exhibits a notable structural variation. While the coordination remains bidentate, the nitro group replaces selenium as one of the donor atoms. This structural shift alters the electronic distribution and may influence these specific complexes’ binding strength and inhibitory potential.

The DFT results support the hypothesis that the bidentate coordination mode contributes significantly to the high inhibitory potency observed experimentally. The stability of these complexes is likely enhanced by the electronic interactions facilitated by this coordination pattern, which optimally positions the ligand for effective urease inhibition. As reflected in their HOMO–LUMO distributions, the electronic properties of these complexes further reinforce the potential for strong ligand-enzyme interactions, with possible implications for the design of new urease inhibitors with improved efficiency.

## 3 Conclusions

The synthesis and characterization of benzoylselenoureas **BSU1-BSU11** and benzoylthioureas **BTU1-BTU11** yielded structurally well-defined compounds, with benzoylselenoureas demonstrating superior inhibitory effects on *C. neoformans* growth and urease activity. The **BSU** exhibited potent antifungal activity, with low MIC and MFC values and remarkable urease inhibition in fungal cells and total protein extracts. The experimental results reveal that the presence of selenium enhances antifungal and antiureolitic potential compared to its sulfur-containing counterparts. Molecular modeling suggests that the selected **BSU** compounds possess a strong affinity for urease, with the potential to act as effective inhibitors. In addition to MD results, the DFT calculations provided key insights into the stability of selenium-containing urease inhibitors by suggesting that their predominant coordination mode is bidentate, possibly involving the amide oxygen, selenium, and Ni­(II). This coordination pattern enhances orbital overlap and electronic interactions, contributing to the strong binding affinity observed. The HOMO–LUMO analysis further supports the role of electronic distribution in stabilizing these complexes. The observed structural variation in **BSU10**, where the selenium atom is replaced by the nitro group as the coordination center, highlights the impact of donor selection on electronic properties and binding strength. These results indicate that electronic and structural factors likely drive the stability of these inhibitors, offering valuable guidance for designing more effective urease inhibitors. The interplay between Ni coordination, hydrogen bonding, and hydrophobic interactions collectively enhances binding stability, reinforcing the potential of these complexes for further experimental validation. These findings reinforce the importance of urease as a promising target in antifungal chemotherapy and highlight the potential of selenium-based compounds in developing new treatments for cryptococcosis.

## 4 Methods

### 4.1 Synthesis of Benzoylselenoureas and Benzoylthioureas

Benzoylselenoureas **BSU1-BSU11** were synthesized according to the protocol described previously^16^ with modifications (Scheme 1). The detailed protocol is described in the Supporting data. The final products were characterized by ^1^H (400 MHz), ^13^C (100 MHz), and ^77^Se (51.5 MHz referenced to external C­(NH_2_)_2_Se) NMR, FTIR-ATR spectra were recorded in the range of 4000–400 cm^–1^, and High-Resolution Mass Spectrometry (IT-TOF). Benzoylthioureas **BTU1-BSU11** analogs were synthesized and characterized as previously reported.^10^

### 4.2 Antifungal Activity of BSU and BTU on *C. neoformans*

*C. neoformans* H99 (or ATCC 208821), a standard strain, was used to evaluate the antifungal activity of **BSU1-BSU11** and **BTU1-BTU11** compounds using the broth microdilution method.^21^ The assay was performed in two different culture media: the standard medium RPMI 1640 buffered with 0.165 MOPS (RMPI), and the Christensen broth (urea medium: 1 g/L peptone, 1 g/L dextrose, 5 g/L sodium chloride, 2 g/L potassium phosphate, 20 g/L urea, 12 mg/L phenol red, pH 7.0). The compounds were dissolved in dimethyl sulfoxide (DMSO) and diluted in both media, obtaining final concentrations from 0.03 to 128 mg/L in a 96-well plate and final DMSO concentration below 0.5%, considered nontoxic. For assay, yeasts were distributed in each well to obtain a final inoculum of 0.5–2.5 × 10^3^ CFU/mL, and the plate was incubated at 35 °C for 48 h.

The MIC values were determined in both media and were defined as the lowest concentrations of **BSU1-BSU11** and **BTU1-BTU11** that inhibited 50% of fungal growth visually compared with untreated yeasts (growth control). In addition, the urease activity was indicated by medium color change from orange to pink due to ammonia production and the MIC_ure_ was defined as the lowest concentration that inhibited the urease activity by visual inspection in urea medium with no color changes. For both assays, two controls were included: untreated yeast group for observation of fungal growth pattern or intracellular urease activity, and wells containing only medium for assay sterility control.

After MIC readings, the MFC values were determined by subculturing, at 35 °C for 48 h, of all concentrations that inhibited the fungal growth; and then MFC was defined as the lowest concentration of the compound capable of reducing ≥99% viability of the initial fungal inoculum.^22,23^

### 4.3 Inhibition of *C. neoformans* Urease Activity

*C. neoformans* H99 yeasts were cultivated in 100 mL of Christensen broth for 72 h at 35 °C by shaking at 200 rpm for the obtention of urease as previously described.^17^ Briefly, the yeasts were ruptured using a lysis buffer (40 mM Tris-HCl, 20 mM dithiothreitol, 4% Triton X-100, 1 mM ethylenediaminetetraacetic acid, 2 mM phenylmethylsulfonyl fluoride, protease inhibitor cocktail) and an ultrasonic sonicator. After centrifugation, the soluble fraction was dialyzed in a dialysis membrane submerged in 10 mM sodium phosphate buffer, pH 7.0; the sample was lyophilized and stored at −20 °C. Before the assay, the sample was resuspended in 1 mL of sodium phosphate buffer (10 mM, pH 7.0), and the total protein was quantified by the Bradford method.^32^

The urease activity was determined by the indophenol method.^24^ In a 96-well microplate, 10 μL of 100 mM urea was added to 45 μL of total protein and 45 μL of **BSU1-BSU11** (concentrations of 0.06–1000 μg/L) in phosphate buffer. The reaction was maintained for 10 min under shaking at 600 rpm, at room temperature. Then, 45 μL of solution A (1% phenol; 0.005% sodium nitroprusside) and 70 μL of solution B (0.5% sodium hydroxide; 0.1% sodium hypochlorite) were immediately added to the wells in the dark and incubated for 5 min at 50 °C under shaking at 600 rpm. Urease activity was quantified by measuring absorbance at 670 nm. The inhibitory concentration capable of inhibiting 50% of urease activity (ureIC_50_) was determined by nonlinear regression calculation using the GraphPad Prism 5.0 software.

### 4.4 Homology Modeling, Molecular Docking, Dynamics Simulations, and DFT Calculations

The homology modeling of *C. neoformans* urease was performed using a sequence retrieved from the UniProt database (UREA_CRYNJ). A template search was conducted in the Protein Data Bank (PDB) using BLAST and HHpred to identify homologous structures with high sequence identity and structural coverage. The selected template (PDB: 4H9M) was aligned with the query sequence, and a three-dimensional model was generated using SwissModel. The *C. neoformans* urease model was constructed using the template alignment approach within ProMod3 (version 3.2.1). For this final step, the constructed model was evaluated based on the root-mean-square deviation (RMSD) value using the PyMOL server and further validated through the Ramachandran plot, which was generated using SwissModel.^25,26^

After obtaining the three-dimensional model of the protein through homology modeling, its native conformation was acquired through preliminary molecular dynamics (MD) simulations for 200 ns using Desmond software. The resulting structure was then compared to the experimental crystallographic structure of Jack Bean urease (PDB: 4H9M) via superimposition using PyMOL. Our research group previously delineated all parameters for the MD simulations.^27,28^

Finally, the three-dimensional structures of benzoylselenoureas­(**BSU1–11**) were designed using ChemDraw Ultra 12 and subsequently subjected to energy minimization through the semiempirical Austin Model 1 (AM1) method, implemented in Spartan 14 V1.1.4. To ensure reproducibility, software versions and build details were meticulously recorded. For molecular docking, Gold 2020 v.1.10.5 was employed, utilizing the CHEMPLP scoring function to assess binding affinities. The binding poses with the lowest energy values were then selected as initial conformations for 200 ns molecular dynamics (MD) simulations of the protein–ligand complexes, following previously established protocols^27,28^

The DFT-D3 calculations were performed using cluster models that included residues and selenoureas molecules positioned within a 4.0 Å radius of the nickel ion in the structures obtained from MD simulations. These calculations employed the ωB97X-D3 ^29^ exchange-correlation functional combined with the def2-SVP basis set^30^ and were carried out using the 5.1 version of the ORCA code.^31,32^

## Supplementary Material



## Notes

(1) ^1^H and ^13^C NMR spectra for all synthesized compounds. (2) An evaluation of the antifungal activity of benzoylthioureas (BTU) and benzoylselenoureas (BSU) against *C. neoformans* H99 growth and their inhibitory action on fungal urease. (3) ωB97X-D3/def2-SVP optimized structures and frontier molecular orbitals (HOMO and LUMO) of the predicted urease-inhibitor complexes, obtained via DFT calculations (PDF)
