# Supplementary material for: Benzoylselenoureas: A Novel Dual-Action Inhibitor Targeting Fungal Growth and Urease Activity in Cryptococcus neoformans
Source: ACS Omega. 2025 Sep 15;10(37):43120–8. doi: 10.1021/acsomega.5c06398 (PMC12461339; doi:10.1021/acsomega.5c06398)
Supplement: Supplementary file 1 [file ao5c06398_si_001.pdf]

Supplementary Information

Benzoylselenoureas: A novel dual-action inhibitor targeting fungal growth and urease activity in *Cryptococcus neoformans*

Nathália Evelyn Morais Costa[a], Thayná Lopes Barreto[b], Nathalia Monteiro Lins Freire[c], Júlio Cosme Santos da Silva[d], Thiago Mendonça de Aquino[c], Eduardo E. Alberto[a], Kelly Ishida[b], Ângelo de Fátima[a*]

[a] Department of Chemistry, Institute of Exact Sciences, Universidade Federal de Minas Gerais, Belo Horizonte, MG, Brazil.
[b] Department of Microbiology, Institute of Biomedical Sciences, Universidade de São Paulo, São Paulo, SP, Brazil.
[c] Research Group on Therapeutic Strategies - GPET, Institute of Chemistry and Biotechnology, Universidade Federal de Alagoas, Maceió, AL, Brazil.
[d] Institute of Chemistry and Biotechnology, Universidade Federal de Alagoas, Maceió, AL, Brazil.

*Corresponding Author:
Email: adefatima.geqob@gmail.com (AdF)

EXPERIMENTAL SECTION

Synthesis and Characterization

Benzoyl chloride (1 mmol) was added to potassium selenocyanate (1 mmol) in the dark, under argon atmosphere and solvent-free conditions. The reaction mixture was heated under 50 °C for 30 minutes and then cooled to room temperature. Then, a dry acetone solution of suitable aromatic amine (1 mmol) was added to this mixture, stirring under reflux for 5 hours. The reaction mixture was then concentrated in a vacuum, and the pure product was obtained through chromatographic column with dichloromethane.

Spectroscopy data *(*^{1}H, ^{13}C and ^{77}Se NMR spectra of compounds BSU 1-11)

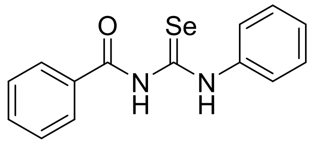 **BSU 1**	**BSU1**: Pale yellow solid, 75% yield. Mp: 137-139°C. IR (ATR, cm $^{-1}$): 3246, 2966, 2920, 1671, 1607, 1597, 1560, 1519, 1485, 1447, 1357, 1300, 1260, 1151, 1131, 1079, 1062, 1025, 999, 982 905, 838, 794, 754, 693, 680, 634, 601, 572. ^{1}H NMR (400 MHz, $CDCl_3$), δ (ppm) = 7.35 (s, 1H), 7.45 (s, 1H), 7.56 (s, 2H), 7.66-7.74 (d, 2H, *J* = 8 Hz),7.90-7.92 (d, 2H, *J* = 8 Hz), 9.45 (s, 1H), 13.01 (s, 1H). ^{13}C NMR (100 MHz, $CDCl_3$), δ (ppm) = 180.2, 167.0, 138.58, 134.1, 131.4, 129.4, 129.1, 127.8, 127.7, 124.8. ^{77}Se NMR (51.5 MHz, $CDCl_3$), δ (ppm) = 385.5.
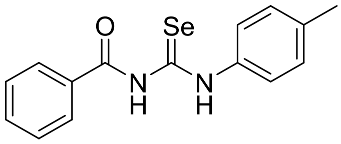 **BSU 2**	**BSU2**: Pale yellow solid, 15% yield. Mp: 127-128°C. IR (ATR, cm $^{-1}$): 3229, 2961, 1667, 1593, 1553, 1516, 1507, 1488, 1352, 1296, 1257, 1154, 1132, 1079, 1065, 999, 938, 832, 815, 698, 653, 620, 596, 574. ^{1}H NMR (400 MHz, $CDCl_3$), δ (ppm) = 2.40 (s, 3H), 7.28-7.29 (d, 2H, *J* = 4 Hz), 7.56-7.60 (t, 4H, *J* = 8 Hz), 7.68-7.72 (t, 1H, *J* = 8 Hz), 7.92-7.95 (d, 2H, *J* = 4 Hz), 9.43 (s, 1H), 12.94 (s, 1H). ^{13}C NMR (100 MHz, $CDCl_3$), δ (ppm) = 180.1, 167.0, 137.7, 136.0, 134.0, 131.4, 129.7, 129.4, 127.7, 124.7, 21.3. ^{77}Se NMR (51.5 MHz, $CDCl_3$), δ (ppm) = 377.9.
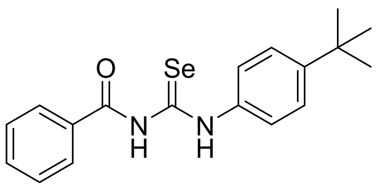 **BSU 3**	**BSU3:** Pale yellow solid, 35% yield. Mp: 210-212°C. IR (ATR, cm $^{-1}$): 2959, 1670, 1599, 1539, 1511, 1488, 1342, 1262, 1131, 1081, 841, 704, 615, 576. ^{1}H NMR (400 MHz, $CDCl_3$), δ (ppm) = 1.34 (s, 9H), 7.45-7.47 (d, 2H, *J* = 8 Hz), 7.54-7.57 (t, 2H, *J* = 8 Hz), 7.65-7.67 (d, 3H, *J* = 8 Hz), 7.90-7.92 (d, 2H, *J* = 8 Hz), 9.44 (s, 1H), 12.98 (s, 1H). ^{13}C NMR (100 MHz, $CDCl_3$), δ (ppm) = 179.6, 167.0, 150.79, 135.8, 134.0, 131.4, 129.4, 127.7, 126.0, 124.1, 34.8, 31.4. ^{77}Se NMR (51.5 MHz, $CDCl_3$), δ (ppm) = 377.8.
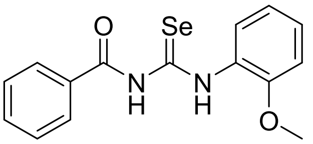 **BSU 4**	**BSU4:** Pale yellow solid, 67% yield. Mp: 150-152°C. IR (ATR, cm $^{-1}$): 3361, 1682, 1602, 1588, 1496, 1456, 1431, 1339, 1277, 1147, 1069, 1029, 861, 782, 741, 695, 611, 552. ^{1}H NMR (400 MHz, $CDCl_3$), δ (ppm) = 3.95 (s, 3H), 6.99 (s, 1H), 7.04 (s, 1H), 7.54 (s, 2H), 7.65 (s, 1H), 7.91 (s, 2H), 8.79 (s, 1H), 9.40 (s, 1H), 13.25 (s, 1H). ^{13}C NMR (100 MHz, $CDCl_3$), δ (ppm) = 177.6, 166.6, 151.2, 133.9, 131.6, 129.3, 127.8, 127.7, 123.6, 120.2, 111.0, 56.1. ^{77}Se NMR (51.5 MHz, $CDCl_3$), δ (ppm) = 393.2.

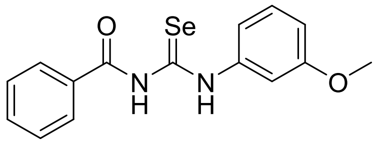<br>**BSU 5**	**BSU5:** Pale yellow solid, 25% yield. Mp: 119-121°C. IR (ATR, cm $^{-1}$): 3387, 2965, 1651, 1611, 1510, 1484, 1451, 1381, 1276, 1207, 1161, 1125, 1026, 1003, 996, 832, 811, 793, 778, 692, 643, 631, 570. ^{1}H NMR (400 MHz, CDCl$_3$), δ (ppm) = 3.87 (s, 3H), 6.93 (s, 1H), 7.36 (s, 1H), 7.53-7.58 (m, 3H), 7.69 (s, 1H), 7.94 (s, 2H), 9.45 (s, 1H), 13.08 (s, 1H). ^{13}C NMR (100 MHz, CDCl$_3$), δ (ppm) = 179.6, 167.0, 160.0, 139.5, 134.1, 131.3, 129.8, 129.4, 127.6, 116.7, 113.6, 109.9, 55.6; ^{77}Se NMR (51.5 MHz, CDCl$_3$), δ (ppm) = 396.6.
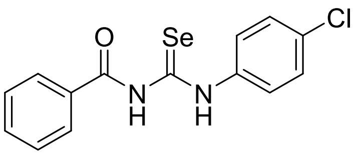<br>**BSU 6**	**BSU6:** Pale yellow solid, 66% yield. Mp: 125-126°C. IR (ATR, cm $^{-1}$): 3196, 3007, 1657, 1587, 1516, 1262, 1138, 1089, 849, 824, 734, 705, 690, 610, 566. ^{1}H NMR (400 MHz, CDCl$_3$), δ (ppm) = 7.39-7.41 (d, 2H, *J* = 8 Hz), 7.54-7.58 (t, 2H, *J* = 8 Hz), 7.66-7.70 (t, 3H, *J* = 12 Hz), 7.89-7.91 (d, 2H, *J* = 12 Hz), 9.48 (s, 1H), 13.03 (s, 1H). ^{13}C NMR (100 MHz, CDCl$_3$), δ (ppm) = 180.5, 167.1, 137.0, 134.2, 133.1, 131.2, 129.4, 129.3, 127.7, 126.0. ^{77}Se NMR (51.5 MHz, CDCl$_3$), δ (ppm) = 397.4.
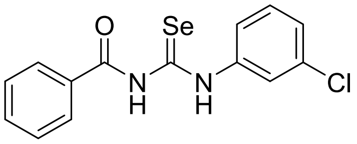<br>**BSU 7**	**BSU7**: Pale yellow solid, 56% yield. Mp: 121-123°C. IR (ATR, cm $^{-1}$): 3284, 2922, 1665, 1586, 1548, 1519, 1471, 1429, 1342, 1254, 1129, 1077, 1024, 871, 792, 708, 689, 623, 570. ^{1}H NMR (400 MHz, CDCl$_3$), δ (ppm) = 7.31-7.33 (d, 1H, *J* = 8 Hz) 7.35-7.39 (t, 1H, *J* = 8 Hz), 7.55-7.58 (t, 2H, *J* = 8 Hz), 7.61-7.63 (d, 1H, *J* = 8 Hz), 7.67-7.70 (t, 1H, *J* = 8 Hz), 7.84 (s, 1H), 7.90-7.92 (d, 2H, *J* = 8 Hz). ^{13}C NMR (100 MHz, CDCl$_3$), δ (ppm) = 180.5, 167.1, 139.6, 134.7, 134.2, 131.2, 130.1, 129.4, 127.7, 124.8, 122.9. ^{77}Se NMR (51.5 MHz, CDCl$_3$), δ (ppm) = 406.8.
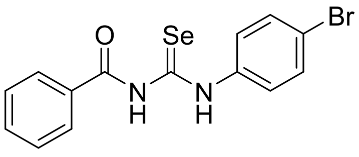<br>**BSU 8**	**BSU8:** Pale yellow solid, 40% yield. Mp: 129-132°C. IR (ATR, cm $^{-1}$): 3002, 1657, 1578, 1516, 1486, 1398, 13130, 1258, 1137, 1068, 1008, 820, 735, 706, 690, 640, 606, 554. ^{1}H NMR (400 MHz, CDCl$_3$), δ (ppm) = 7.55-7.58 (t, 4H, *J* = 8 Hz), 7.63-7.70 (m, 2H), 7.89-7.91 (t, 2H, *J* = 8 Hz), 9.46 (s, 1H), 13.03 (s, 1H). ^{13}C NMR (100 MHz, CDCl$_3$), δ (ppm) = 180.3, 167.1, 137.5, 134.2, 132.3, 131.2, 129.4, 127.7, 126.3, 121.0. ^{77}Se NMR (51.5 MHz, CDCl$_3$), δ (ppm) = 400.0. Agree with the reference 15.
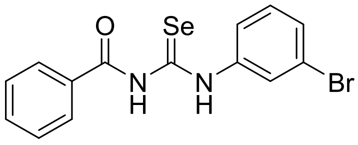<br>**BSU 9**	**BSU9:** Pale yellow solid, 30% yield. Mp: 120-122°C. IR (ATR, cm $^{-1}$): 3330, 2926, 1664, 1574, 1519, 1477, 1334, 1254, 1132, 1073, 1024, 867, 771, 702, 684, 671, 615, 552. ^{1}H NMR (400 MHz, Acetonitrile-*d$_3$*), δ (ppm) = 7.34-7.38 (t, 1H, *J* = 8 Hz), 7.50-7.58 (m, 4H), 7.68-7.72 (t, 1H, *J* = 8 Hz), 7.92-7.94 (d, 2H, *J* = 8 Hz), 8.0 (s, 1H), 9.92 (s, 1H), 12.97 (s, 1H). ^{13}C NMR (100 MHz, Acetonitrile-*d$_3$*), δ (ppm) = 182.6, 169.7, 141.5, 134.7, 132.5, 131.4, 131.1, 129.8, 129.2, 125.4, 122.2. ^{77}Se NMR (51.5 MHz, CDCl$_3$), δ (ppm) = 407.6.

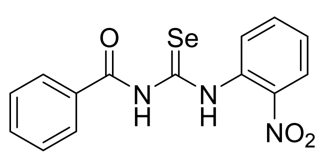 **BSU 10**	**BSU10:** Pale yellow solid, 23% yield. Mp: 140-143°C. IR (ATR, cm $^{-1}$): 3361, 1682, 1602, 1588, 1496, 1456, 1431, 1339, 1252, 1222, 1147, 1069, 1029, 898, 861, 782, 741, 695, 684, 611, 552. ^{1}H NMR (400 MHz, $CDCl_3$), δ (ppm) = 7.13-7.19 (m, 1H), 7.45-7.49 (t, 2H, *J* = 8 Hz), 7.51-7.55 (t, 1H, *J* = 8 Hz), 7.62-7.66 (t, 1H, *J* = 8 Hz), 7.92-7.94 (d, 2H, *J* = 8 Hz), 8.20-8.21 (d, 1H, *J* = 8 Hz), 11.27 (s, 1H). ^{13}C NMR (100 MHz, $CDCl_3$), δ (ppm) = 165.9, 136.6, 136.3, 135.5, 134.1, 132.8, 129.2, 127.5, 123.0, 123.4, 122.2.
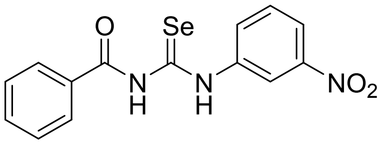 **BSU 11**	**BSU11:** Pale yellow solid, 37% yield. Mp: 190-193°C. IR (ATR, cm $^{-1}$): 3247, 3081, 2924, 1668, 1513, 1341, 1257, 1153, 1133, 1080, 1066, 897, 828, 735, 677, 666, 635, 566. ^{1}H NMR (400 MHz, $CDCl_3$), δ (ppm) = 7.56-7.64 (m, 3H), 7.68-7.72 (t, 1H, *J* = 8 Hz), 7.91-7.94 (t, 2H, *J* = 8 Hz), 8.09-8.12 (d, 1H, *J* = 8 Hz), 8.19-8.22 (d, 1H, *J* = 8 Hz), 8.74 (s, 1H), 9.55 (s, 1H), 13.28 (s, 1H). ^{13}C NMR (100 MHz, $CDCl_3$), δ (ppm) = 181.4, 167.3, 148.5, 139.6, 134.4, 131.0, 130.6, 129.9, 129.5, 127.7, 122.2, 119.9. ^{77}Se NMR (51.5 MHz, $CDCl_3$), δ (ppm) = 423.8.

FIGURES AND TABLES

Table S1. Antifungal activity of benzoylthioureas (**BTU**) and benzoylselenorureas (**BSU**) against *Cryptococcus neoformans* H99 growth and inhibitory action on fungal urease activity.

Compounds	Media	Benzoylthioureas (mg/L)		
		MIC	MIC_{URE}	MFC
**BTU1**	RPMI	>128	ND	>128
	urea	32	32	128
**BTU2**	RPMI	>128	ND	>128
	PEP+	32	≤0.25	>128
**BTU3**	RPMI	8	ND	>128
	urea	1	0.5	128
**BTU4**	RPMI	>128	ND	>128
	urea	1	≤0.25	>128
**BTU5**	RPMI	>128	ND	>128
	urea	4	≤0.25	>128
**BTU6**	RPMI	>128	ND	>128
	urea	32	≤0.25	128
**BTU7**	RPMI	>128	ND	>128
	urea	32	2	64
**BTU8**	RPMI	>128	ND	>128
	urea	64	32	>128
**BTU9**	RPMI	32	ND	>128
	urea	4	4	32
**BTU10**	RPMI	>128	ND	>128

	urea	>128	>128	>128
**BTU11**	RPMI	>128	ND	>128
	urea	>128	>128	>128

**Compounds**	**Media**	**Benzoylselenoureas (mg/L)**		
		**MIC**	**MIC$_{URE}$**	**MFC**
**BSU1**	RPMI	2	ND	2
	urea	0.06	0.06	0.12
**BSU2**	RPMI	16	ND	16
	urea	0.5	0.5	1
**BSU3**	RPMI	>16	ND	>16
	urea	16	16	>16
**BSU4**	RPMI	16	ND	16
	urea	0.5	0.25	0.5
**BSU5**	RPMI	8	ND	16
	urea	0.25	0.12	0.25
**BSU6**	RPMI	>16	ND	>16
	urea	8	8	16
**BSU7**	RPMI	4	ND	4
	urea	0.12	0.06	0.25
**BSU8**	RPMI	8	ND	8
	urea	0.5	0.5	0.5
**BSU9**	RPMI	1	ND	1
	urea	0.12	0.12	0.5
**BSU10**	RPMI	8	ND	16
	urea	1	1	2
**BSU11**	RPMI	16	ND	>16
	urea	1	1	1

MIC, Minimum inhibitory concentration required to inhibit 50% of fungal growth in RPMI or urea media.
MIC$_{ure}$, Minimum inhibitory concentration required to inhibit 100 % yeast urease activity in urea medium.
MFC, Minimum fungicidal concentration values obtained after assay in RPMI medium.

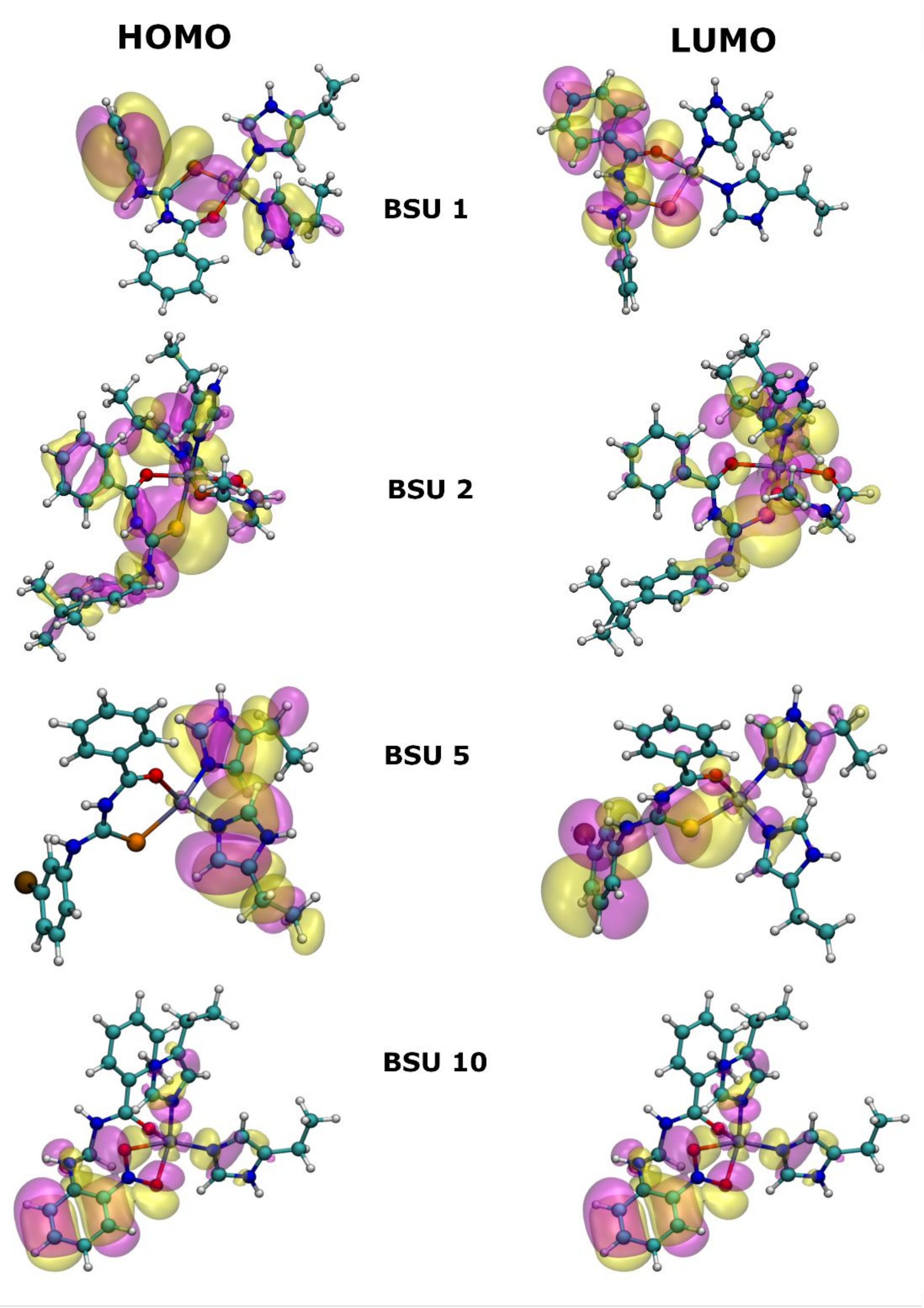

Figure S1. ωB97x-D3/def2-SVP optimized structures and Frontier molecular orbitals (HOMO and LUMO) of the predicted urease-inhibitor complexes obtained via DFT calculations.

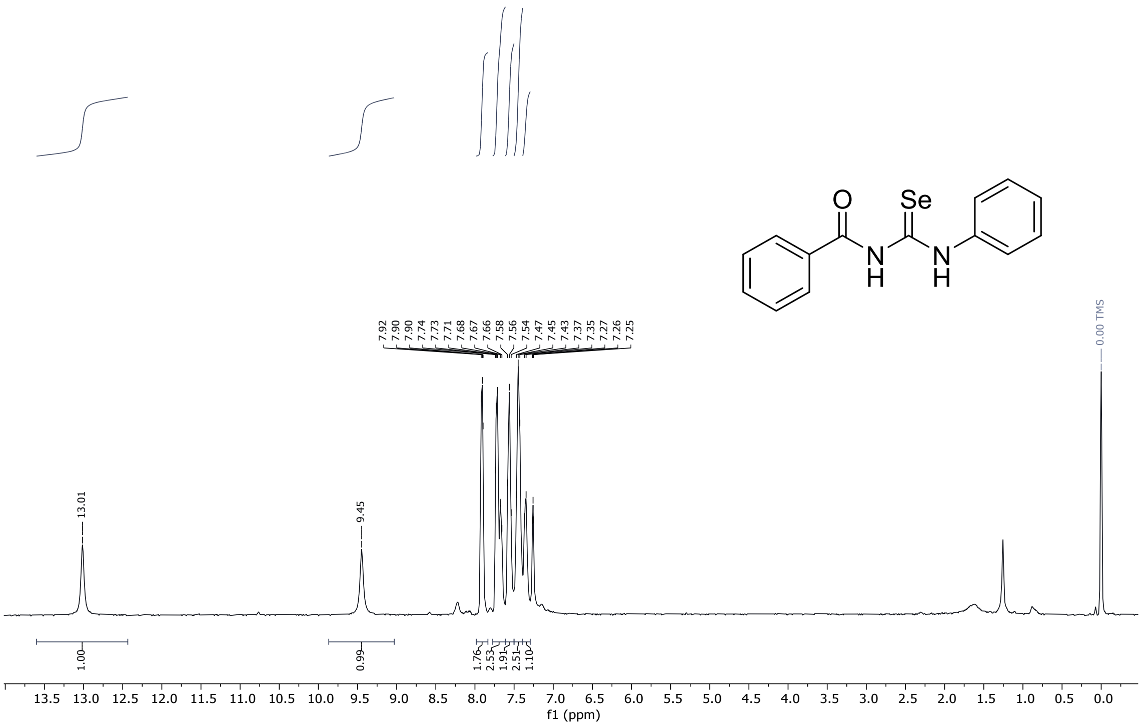

Figure S2: ^{1}H NMR (400 MHz, $CDCl_3$) – compound **BSU1**

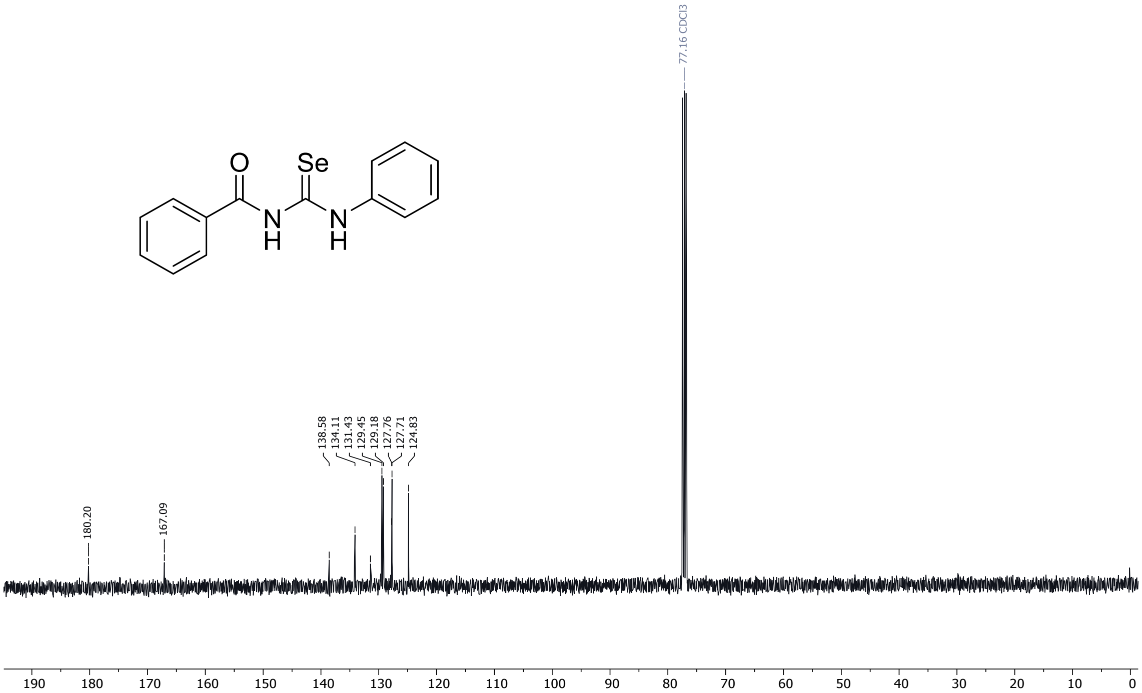

Figure S3: ^{13}C NMR (100 MHz, $CDCl_3$) – compound **BSU1**

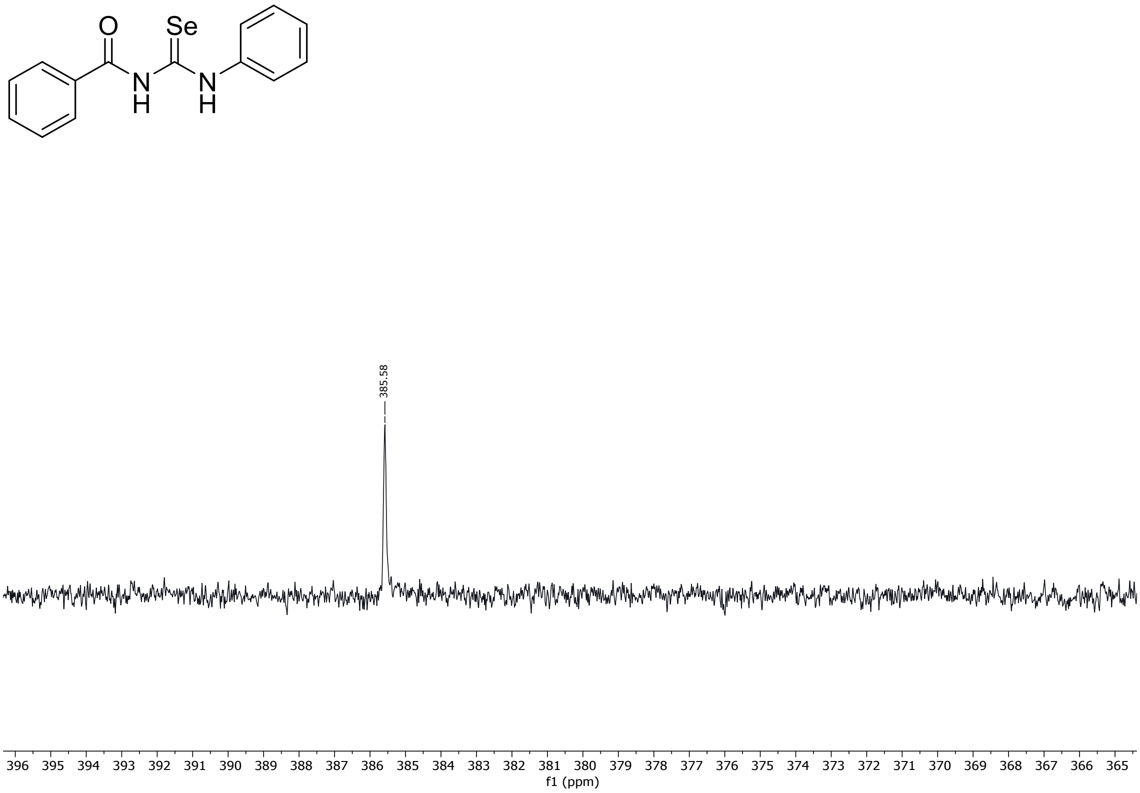

Figure S4: ^{77}Se NMR (51.5 MHz, $CDCl_3$) – compound **BSU1**

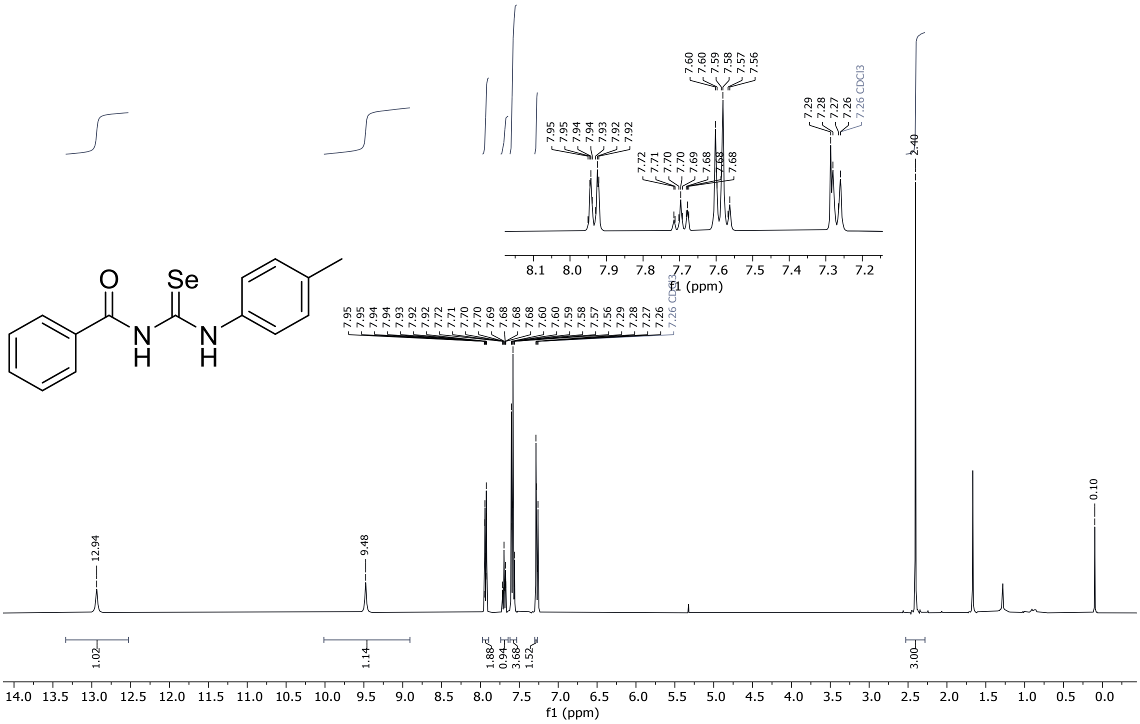

Figure S5: ^{1}H NMR (400 MHz, $CDCl_3$) – compound **BSU2**

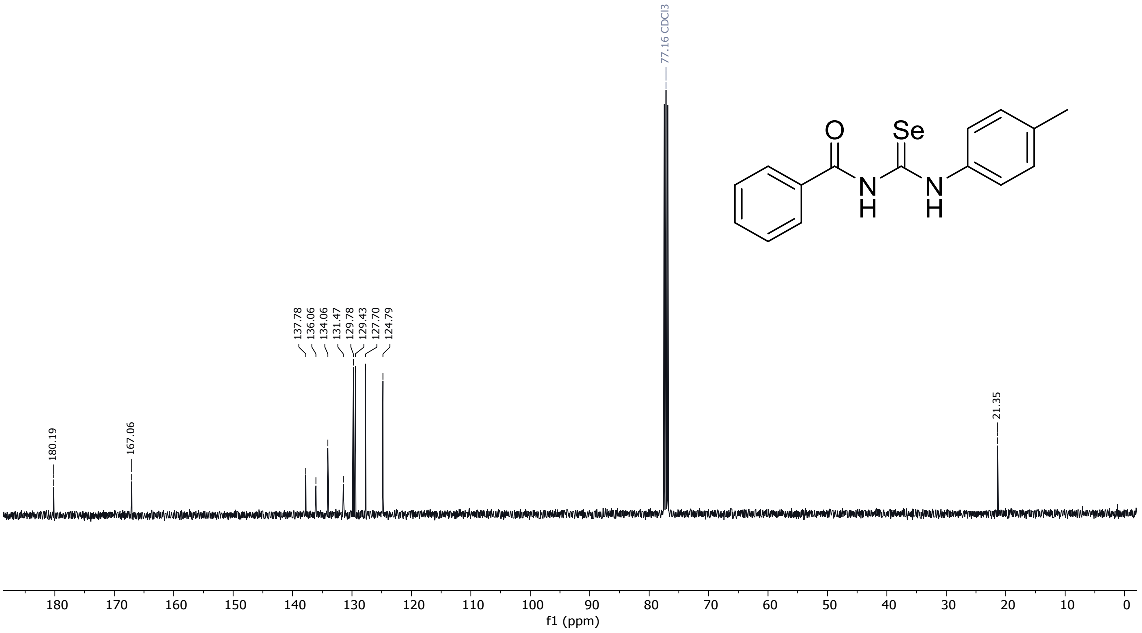

Figure S6: ^{13}C NMR (100 MHz, $CDCl_3$) – compound **BSU2**

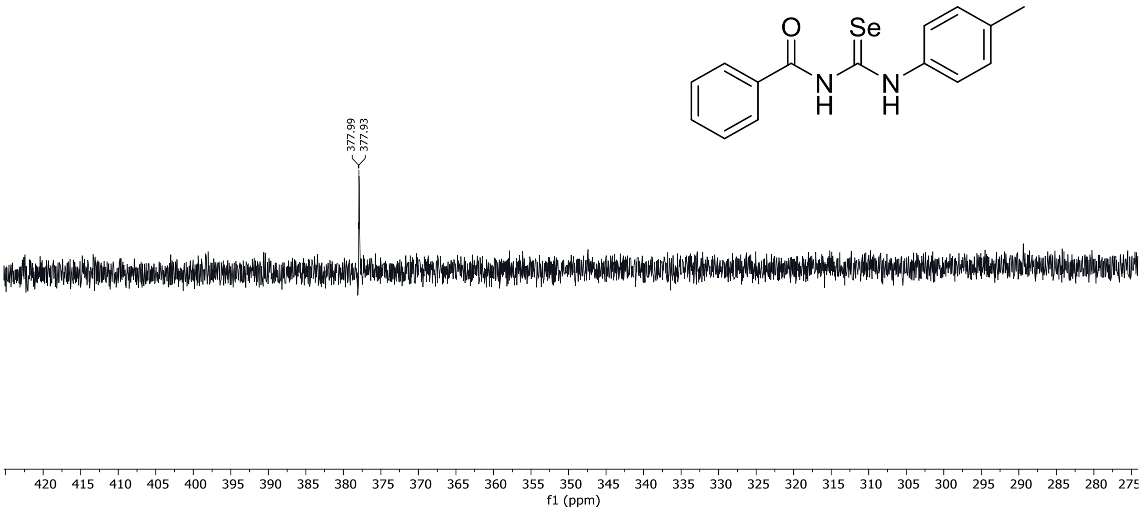

Figure S7: ^{77}Se NMR (51.5 MHz, $CDCl_3$) – compound **BSU2**

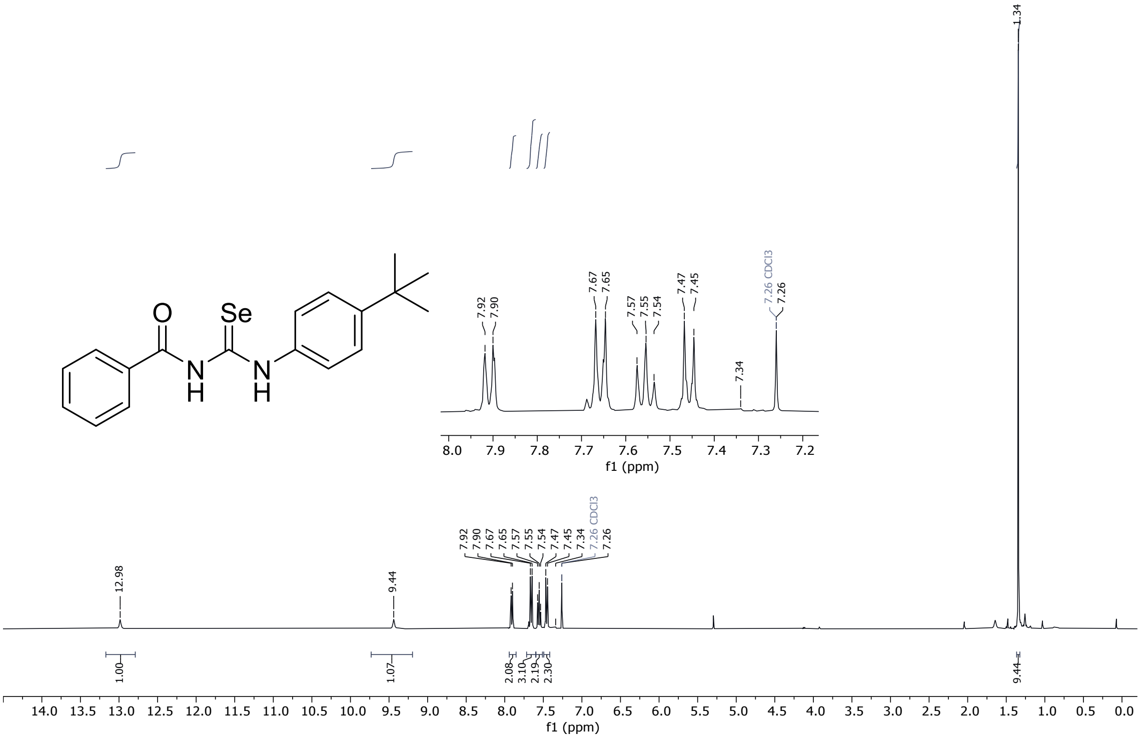

Figure S8: ¹H NMR (400 MHz, $CDCl_3$) – compound **BSU3**

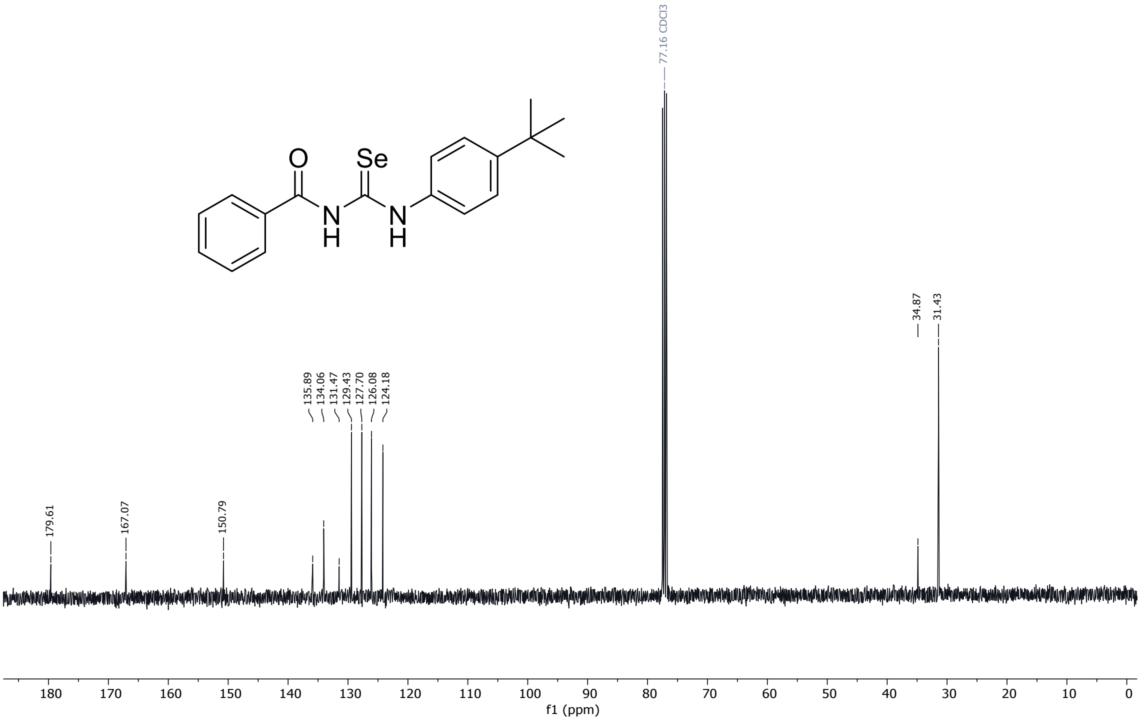

Figure S9: ¹³C NMR (100 MHz, $CDCl_3$) – compound **BSU3**

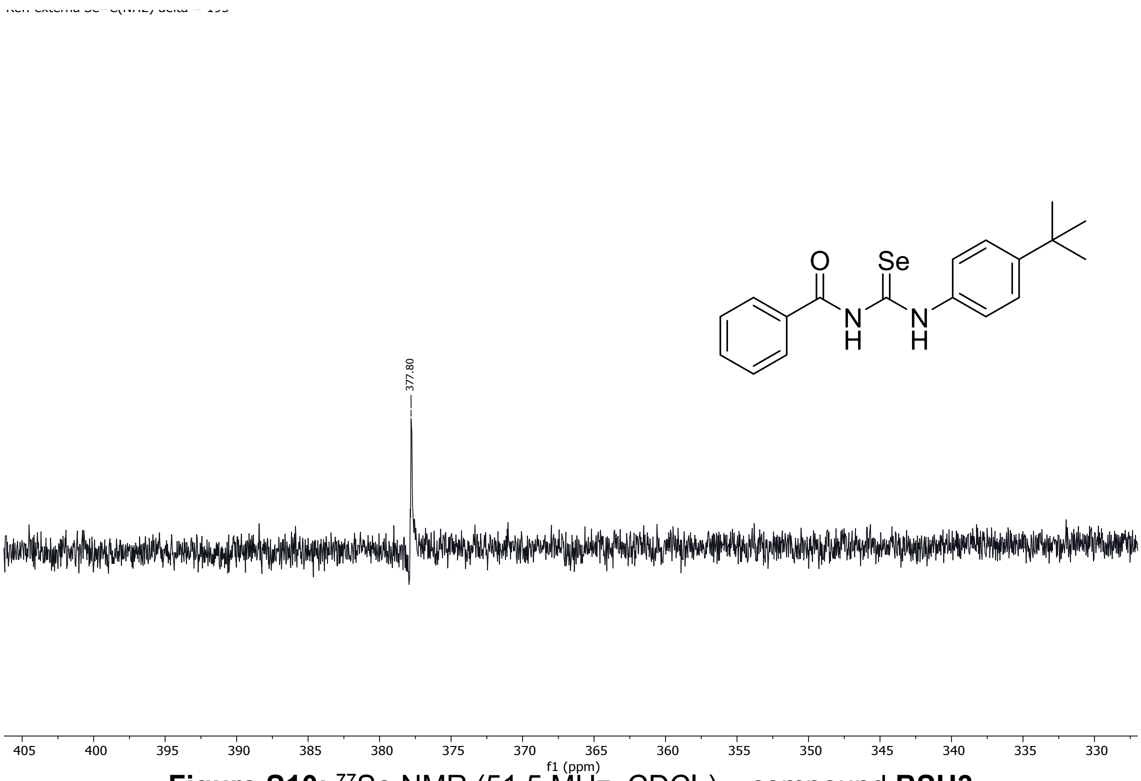

Figure S10: ^{77}Se NMR (51.5 MHz, $CDCl_3$) – compound **BSU3**

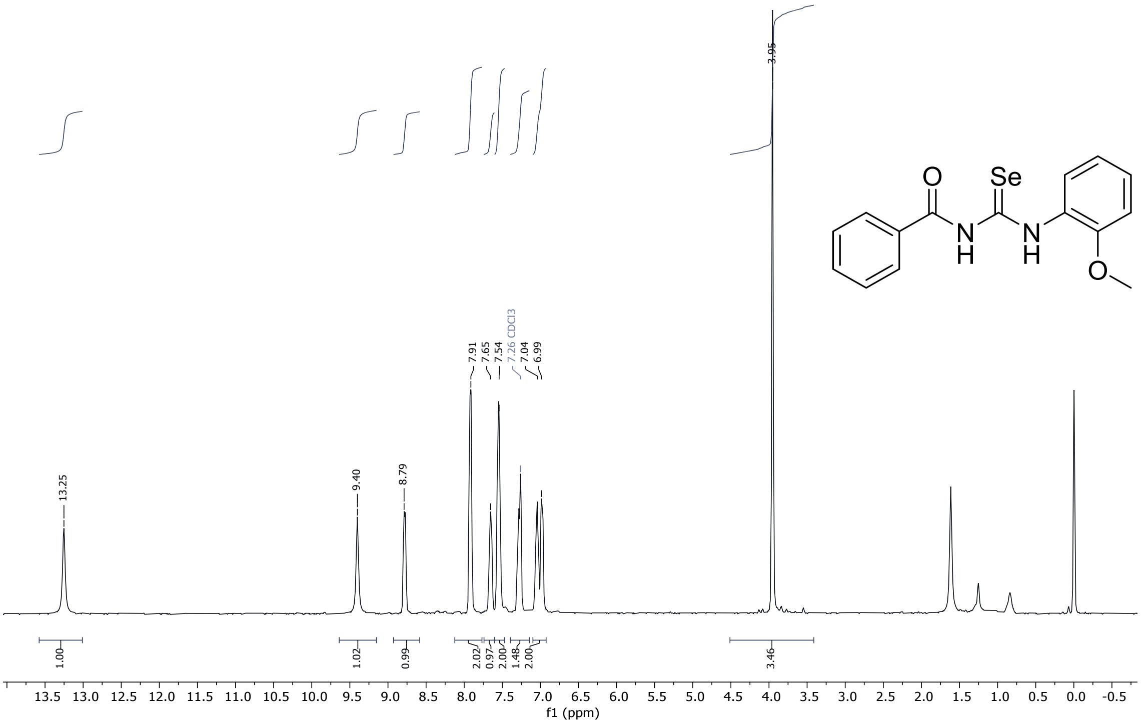

Figure S11: ^{1}H NMR (400 MHz, $CDCl_3$) – compound **BSU4**

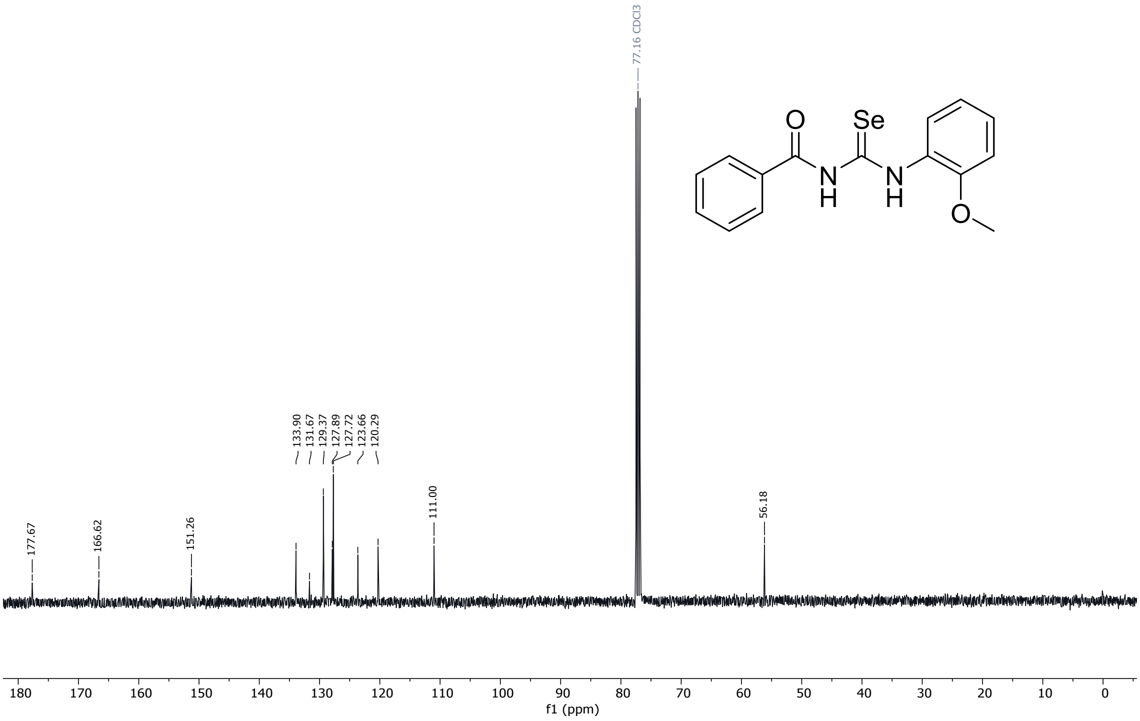

Figure S12: ^{13}C NMR (100 MHz, $CDCl_3$) – compound **BSU4**

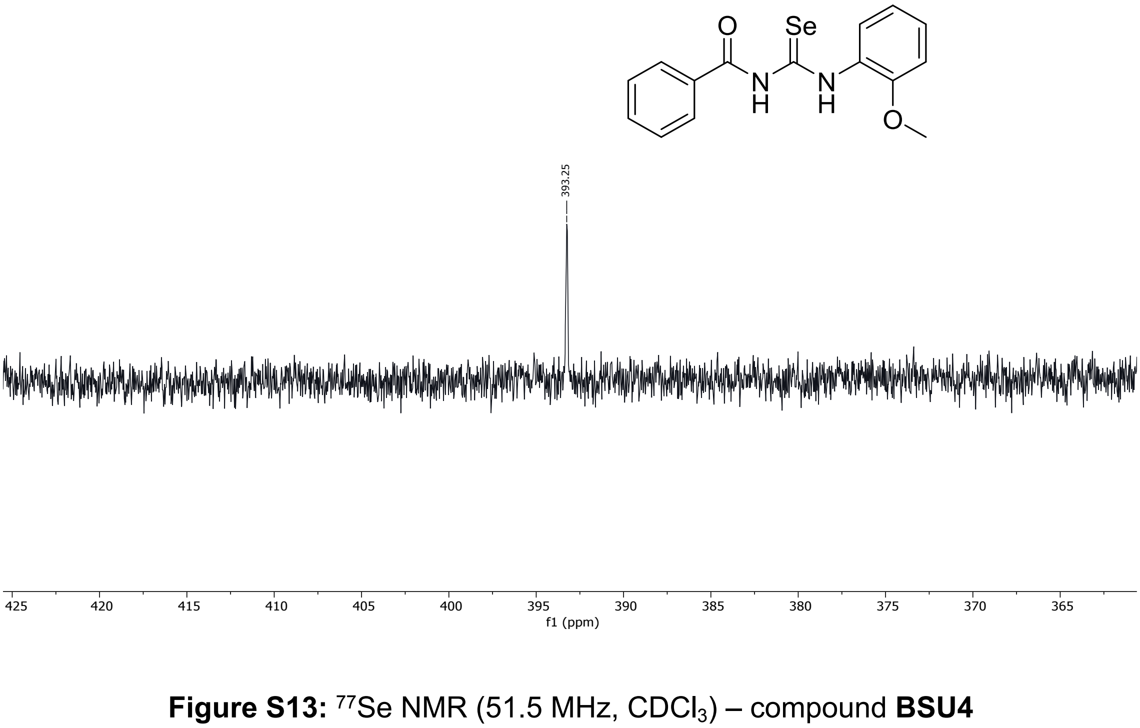

Figure S13: ^{77}Se NMR (51.5 MHz, $CDCl_3$) – compound **BSU4**

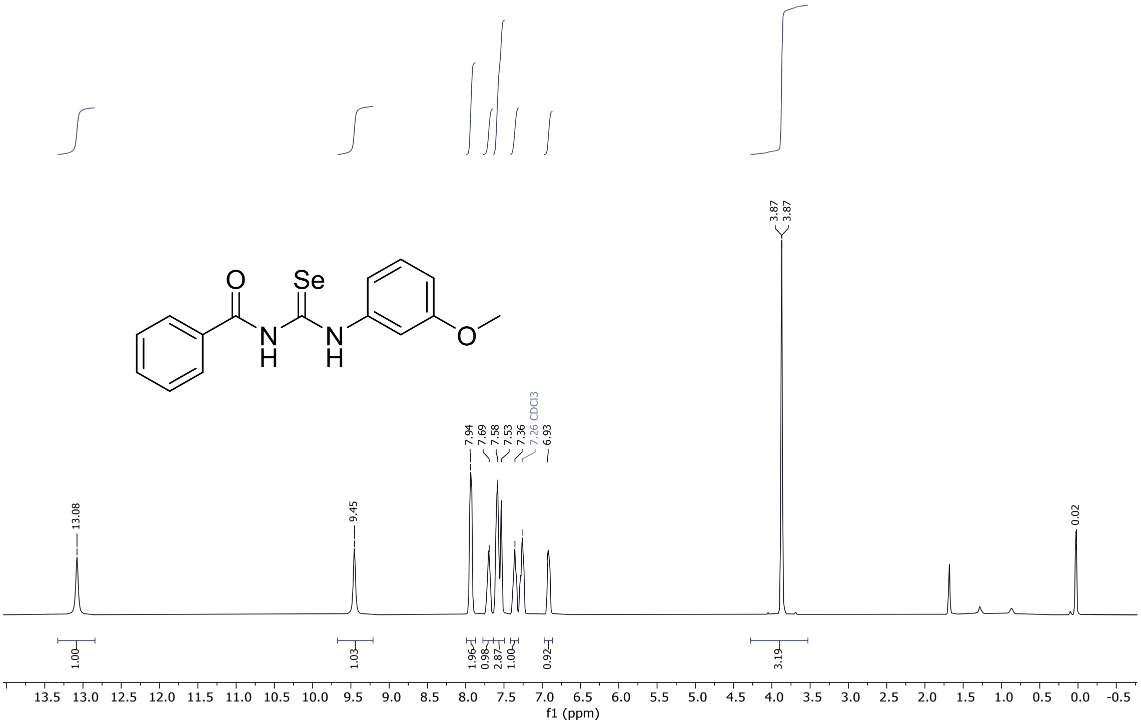

Figure S14: ^{1}H NMR (400 MHz, $CDCl_3$) – compound **BSU5**

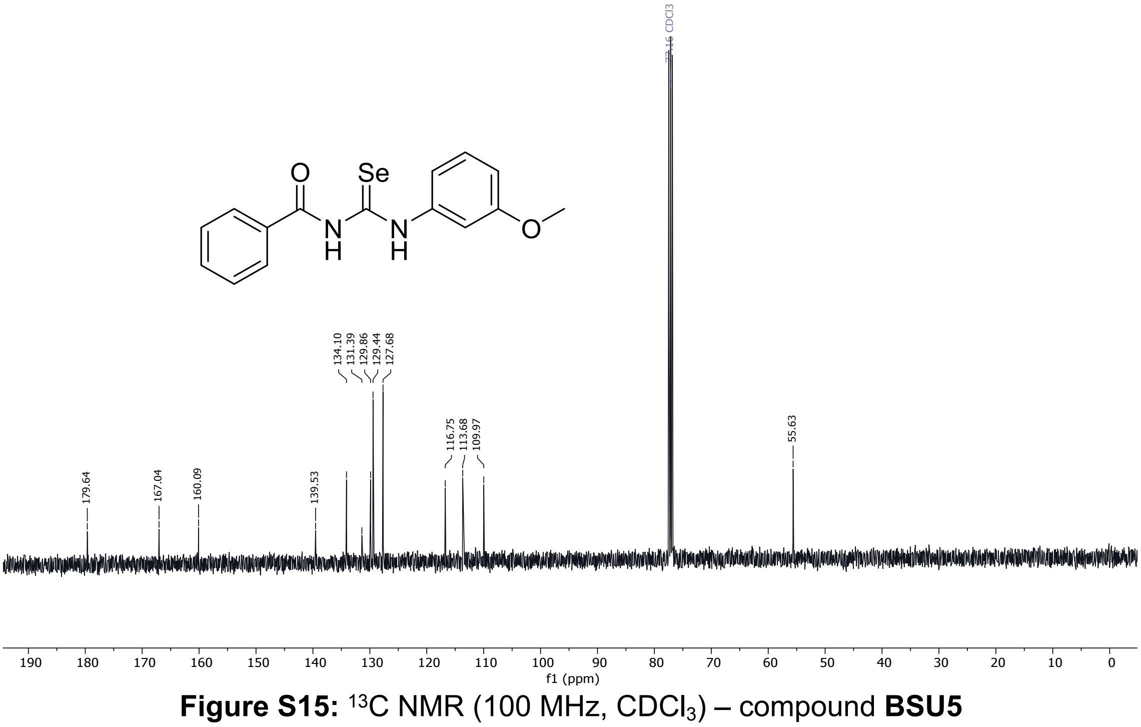

Figure S15: ^{13}C NMR (100 MHz, $CDCl_3$) – compound **BSU5**

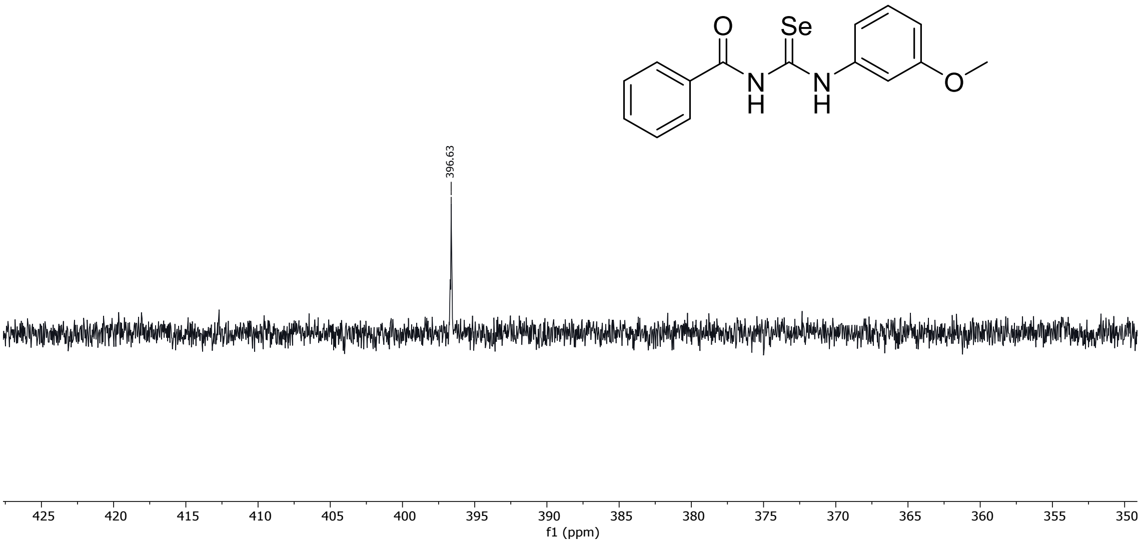

Figure S16: ^{77}Se NMR (51.5 MHz, $CDCl_3$) – compound **BSU5**

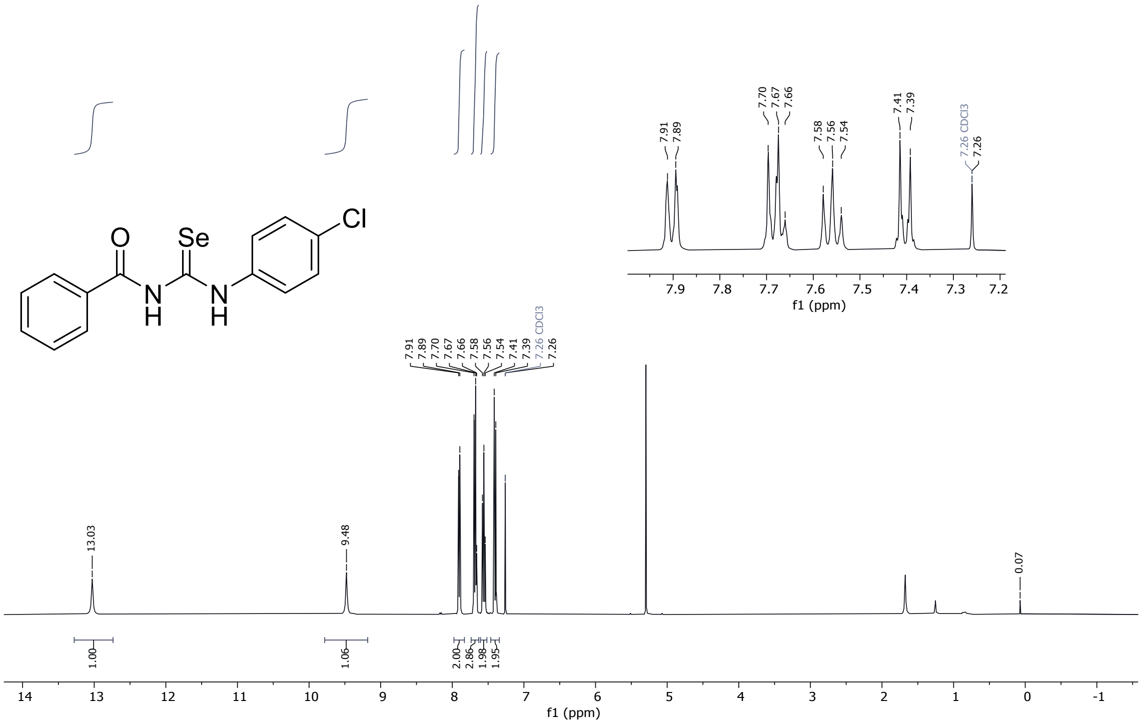

Figure S17: ^{1}H NMR (400 MHz, $CDCl_3$) – compound **BSU6**

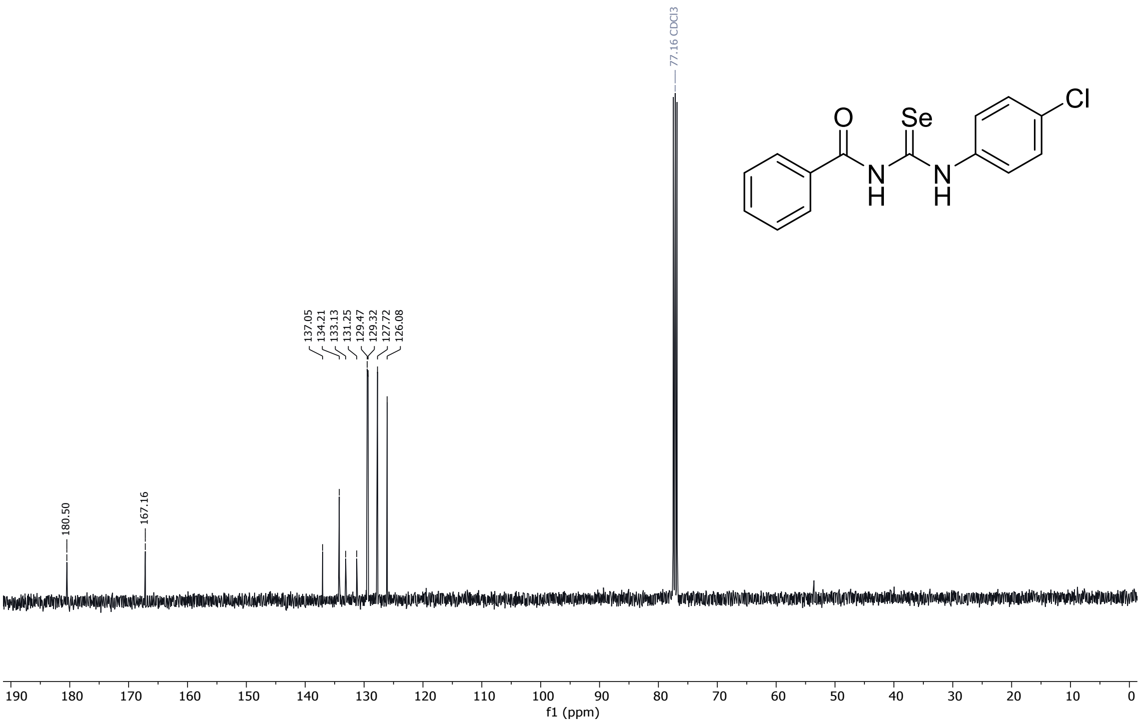

Figure S18: ^{13}C NMR (100 MHz, $CDCl_3$) – compound **BSU6**

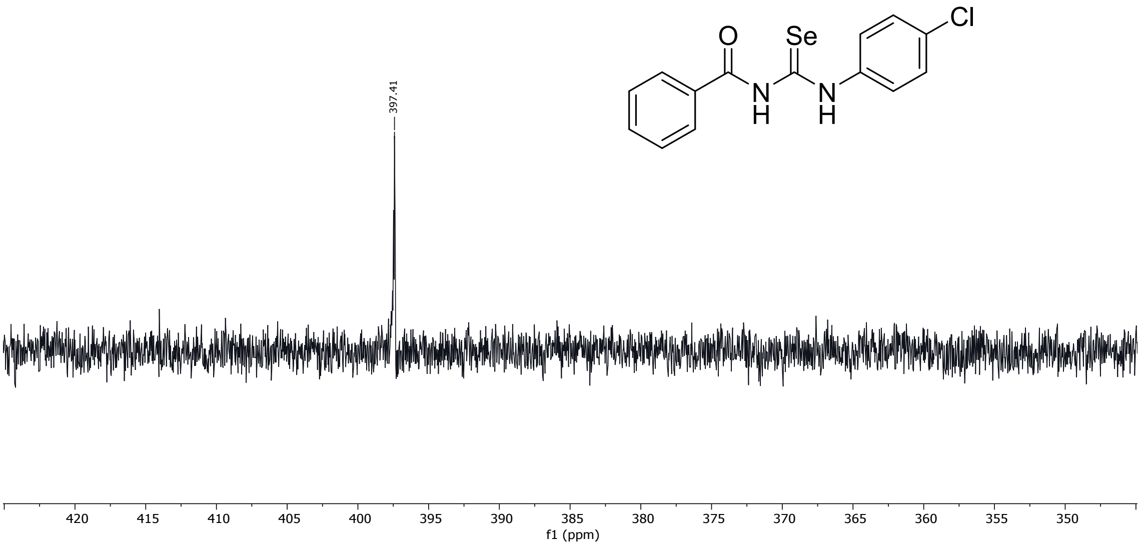

Figure S19: ^{77}Se NMR (51.5 MHz, $CDCl_3$) – compound **BSU6**

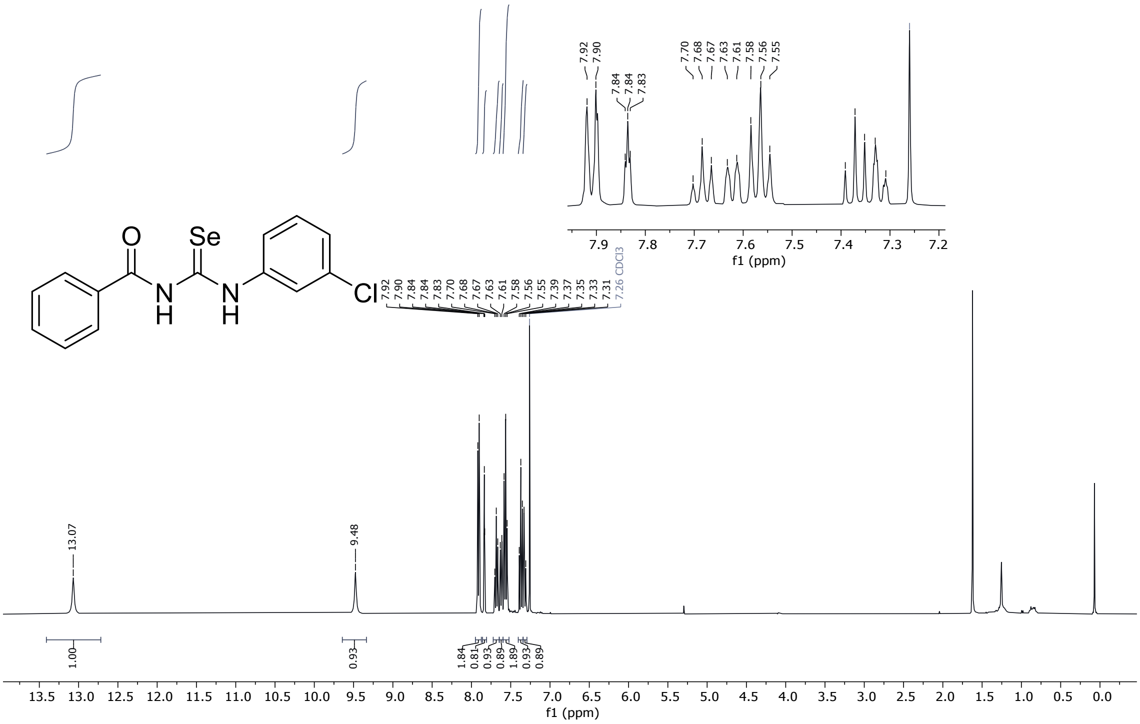

Figure S20: ^{1}H NMR (400 MHz, $CDCl_3$) – compound **BSU7**

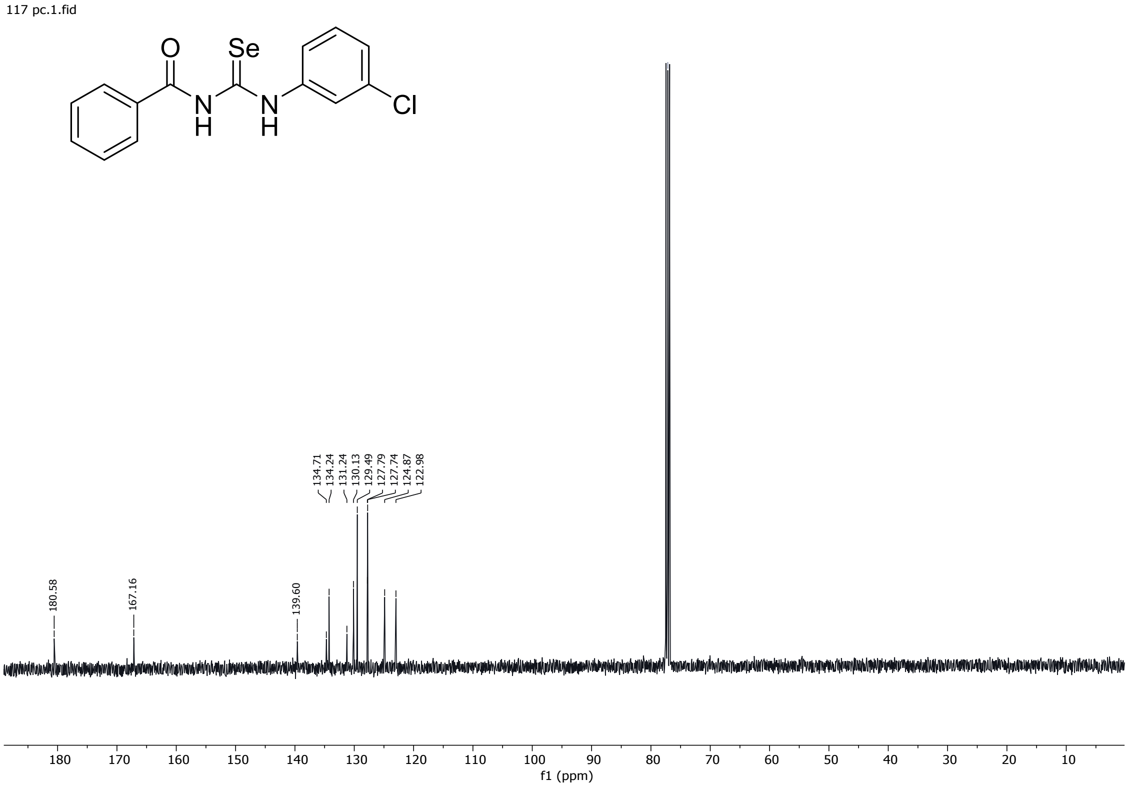

Figure S21 ^{13}C NMR (100 MHz, $CDCl_3$) – compound **BSU7**

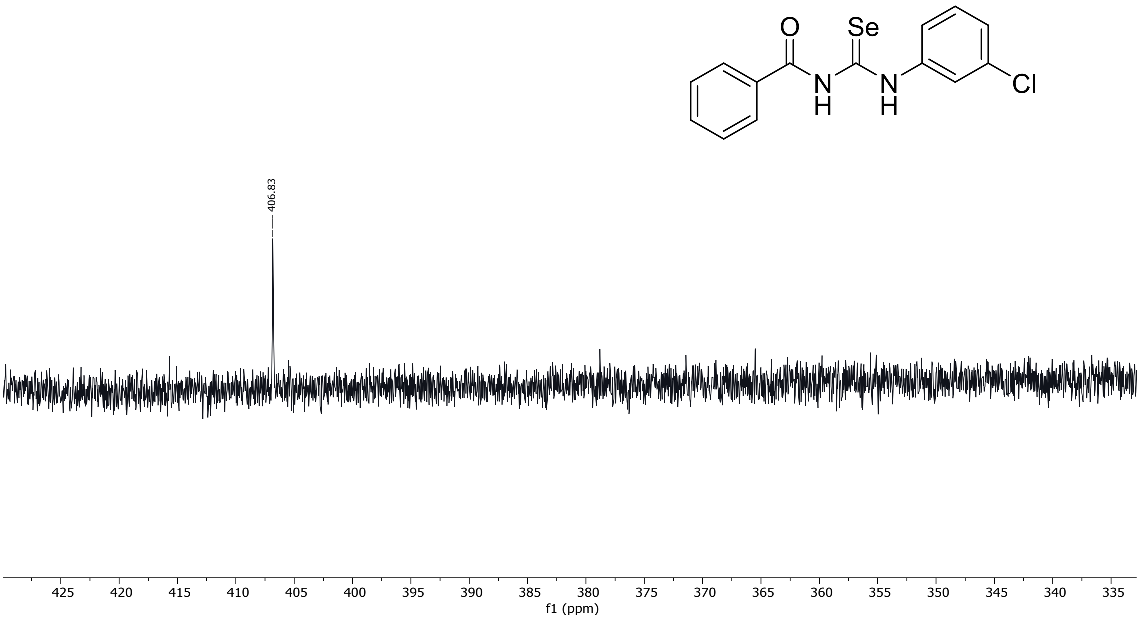

Figure S22: ^{77}Se NMR (51.5 MHz, $CDCl_3$) – compound **BSU7**

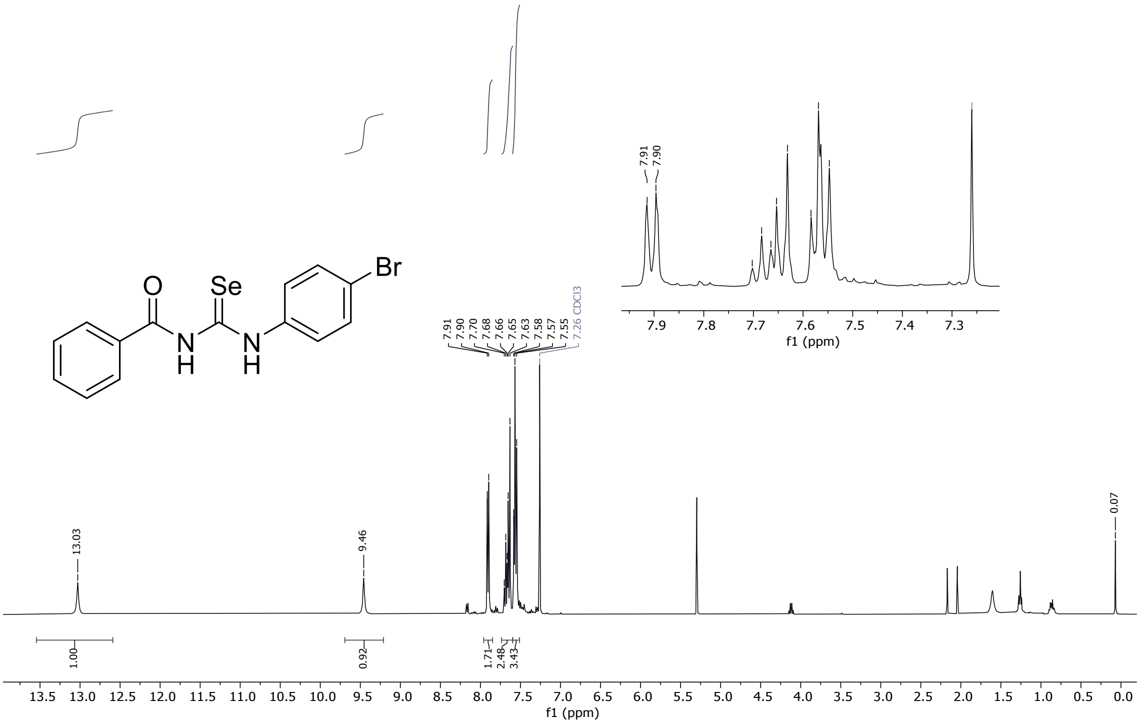

Figure S23: ^{1}H NMR (400 MHz, $CDCl_3$) – compound **BSU8**

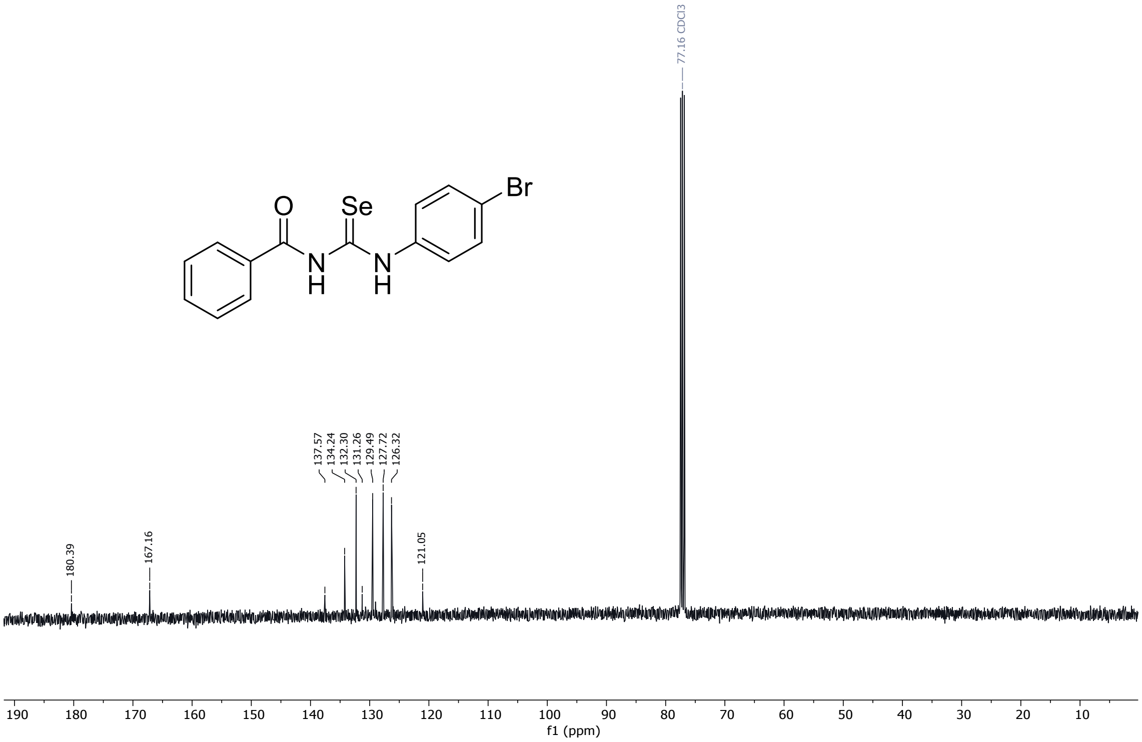

Figure S24: ^{13}C NMR (100 MHz, $CDCl_3$) – compound **BSU8**

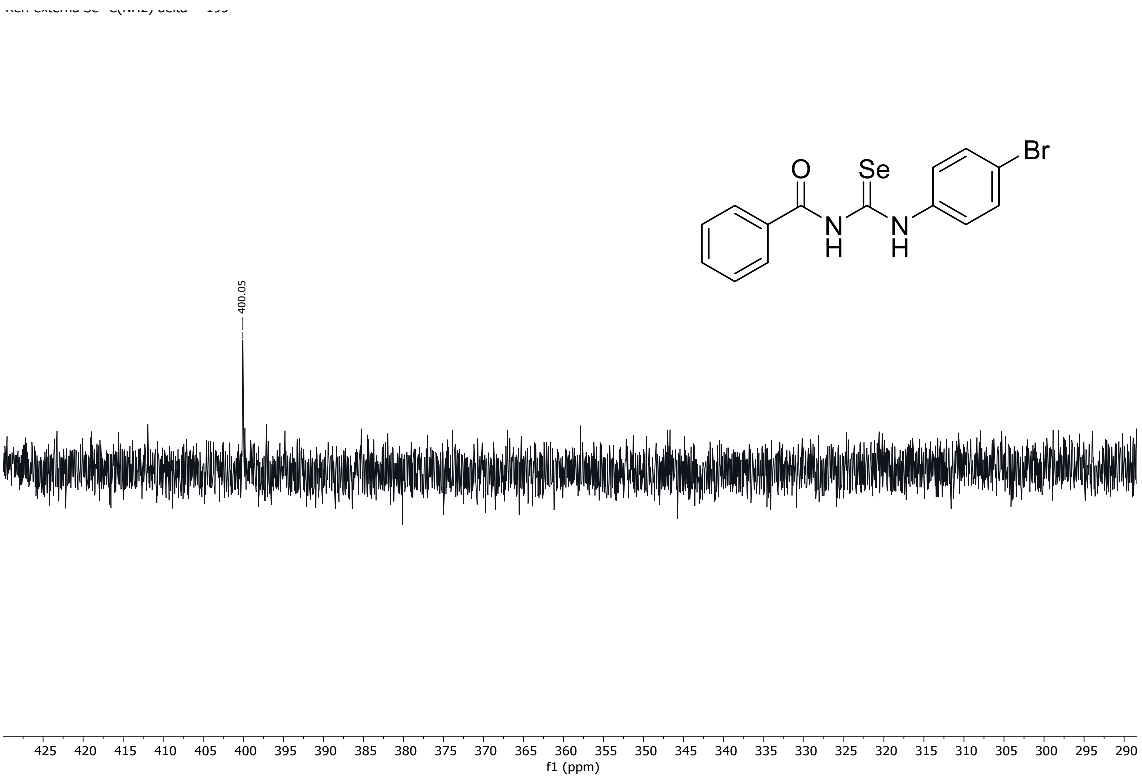

Figure S25: ^{77}Se NMR (51.5 MHz, $CDCl_3$) – compound **BSU8**

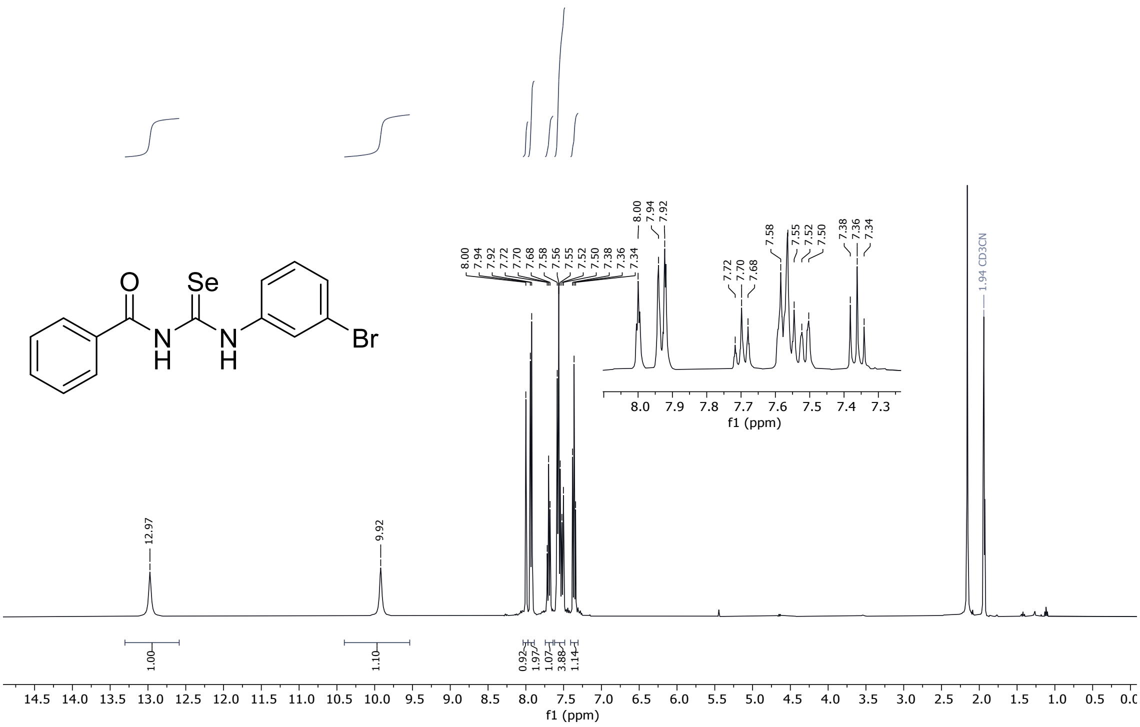

Figure S26: ^{1}H NMR (400 MHz, acetonitrile-d_3) – compound **BSU9**

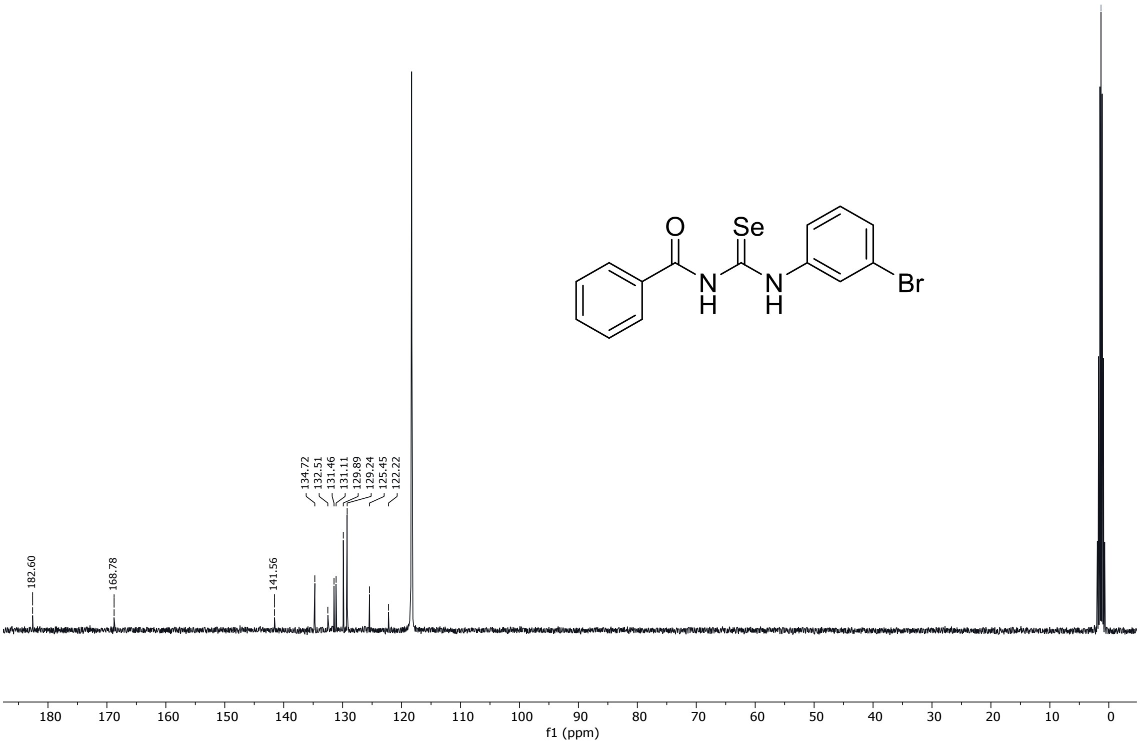

Figure S27: ^{13}C NMR (100 MHz, acetonitrile-d_3) – compound **BSU9**

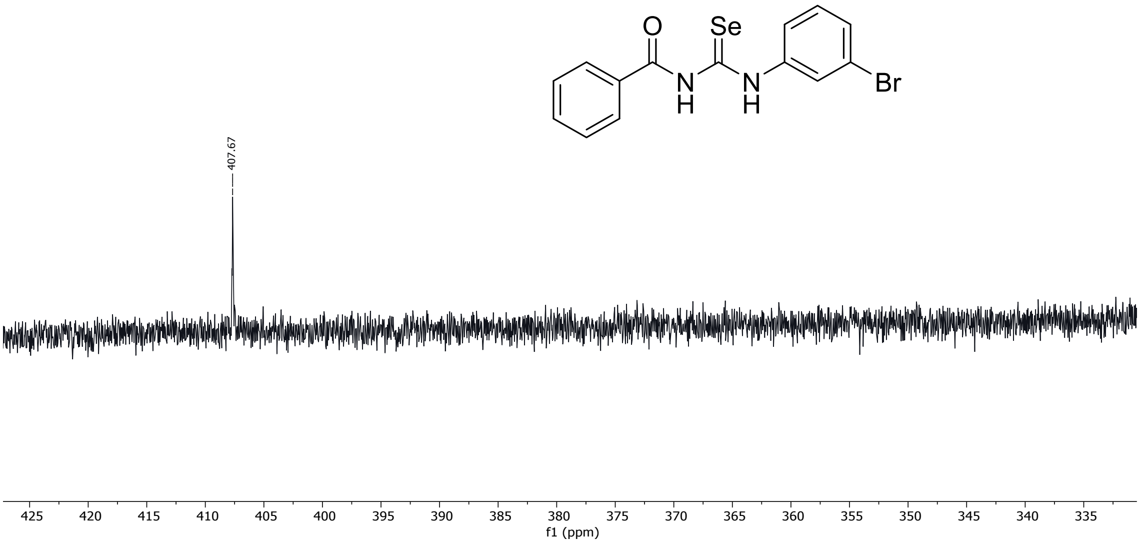

Figure S28: ^{77}Se NMR (51.5 MHz, acetonitrile-d_3) – compound **BSU9**

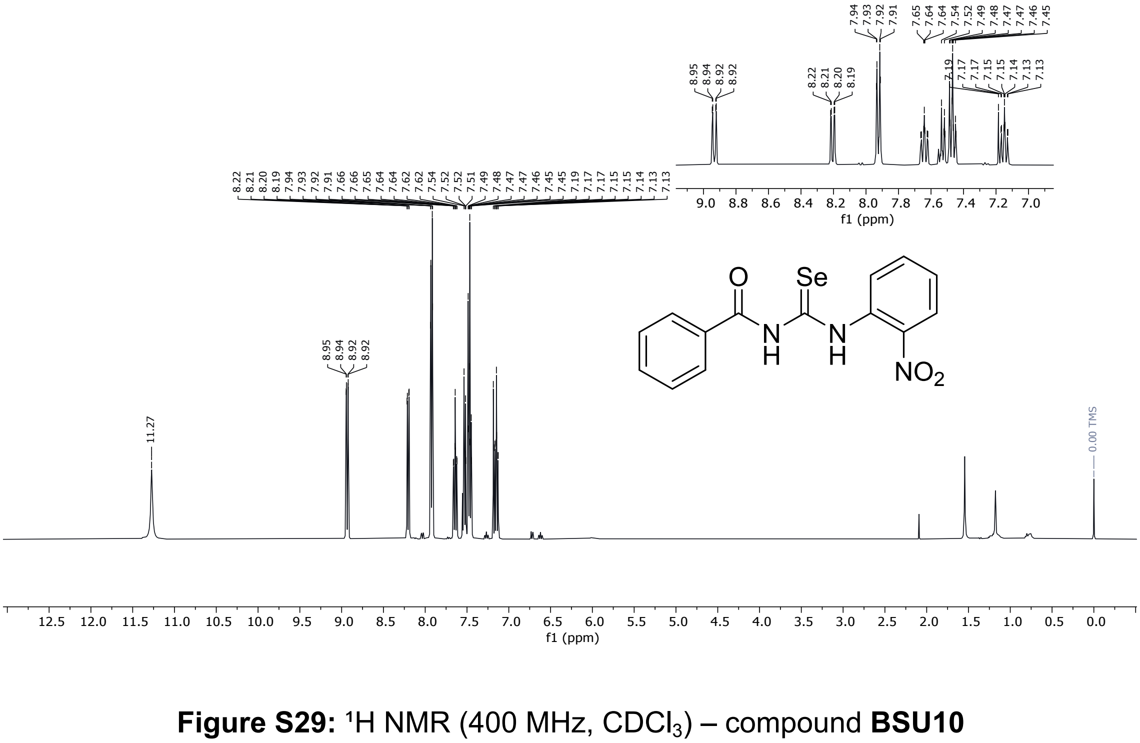

Figure S29: ^{1}H NMR (400 MHz, $CDCl_3$) – compound **BSU10**

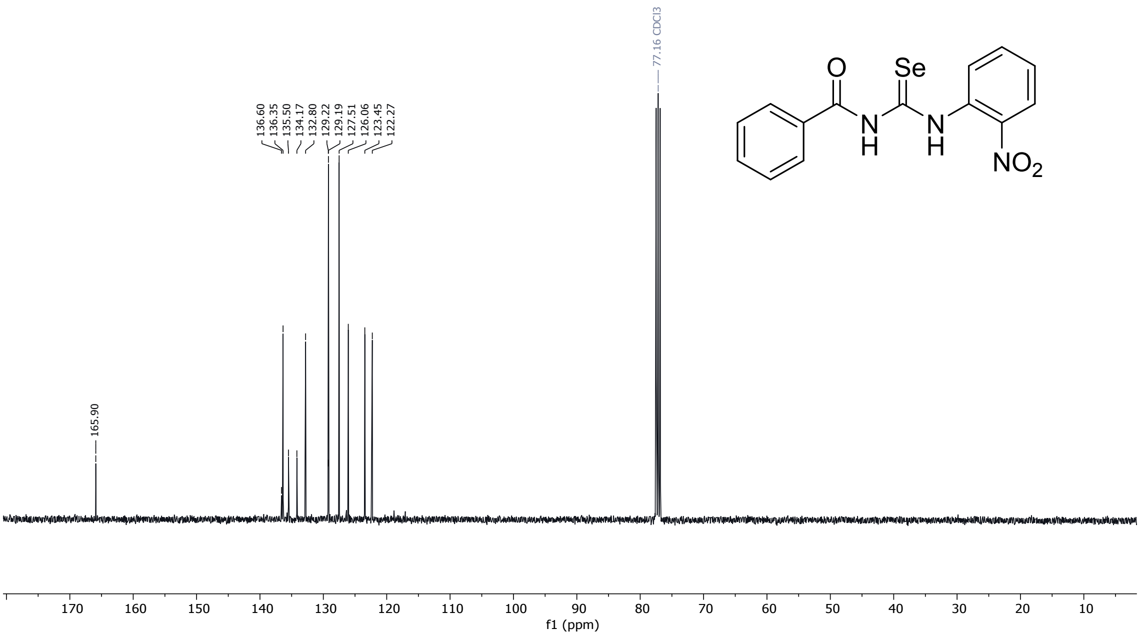

Figure S30: ^{13}C NMR (100 MHz, $CDCl_3$) – compound **BSU10**

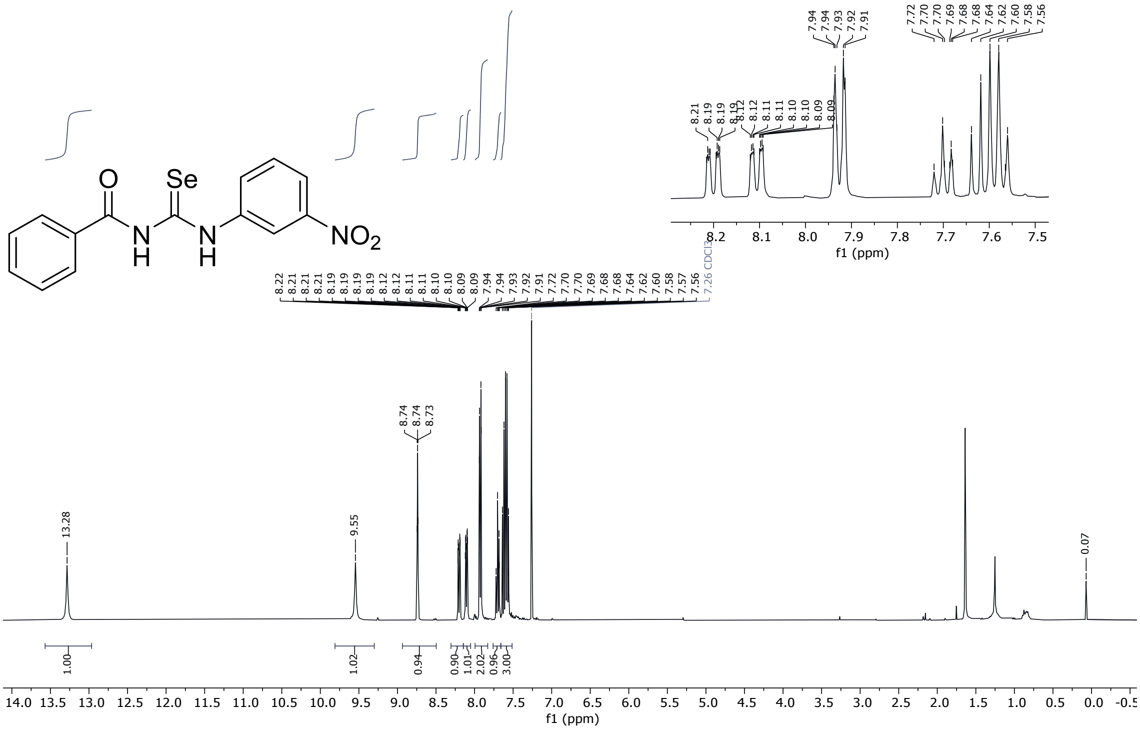

Figure S31: ^{1}H NMR (400 MHz, $CDCl_3$) – compound **BSU11**

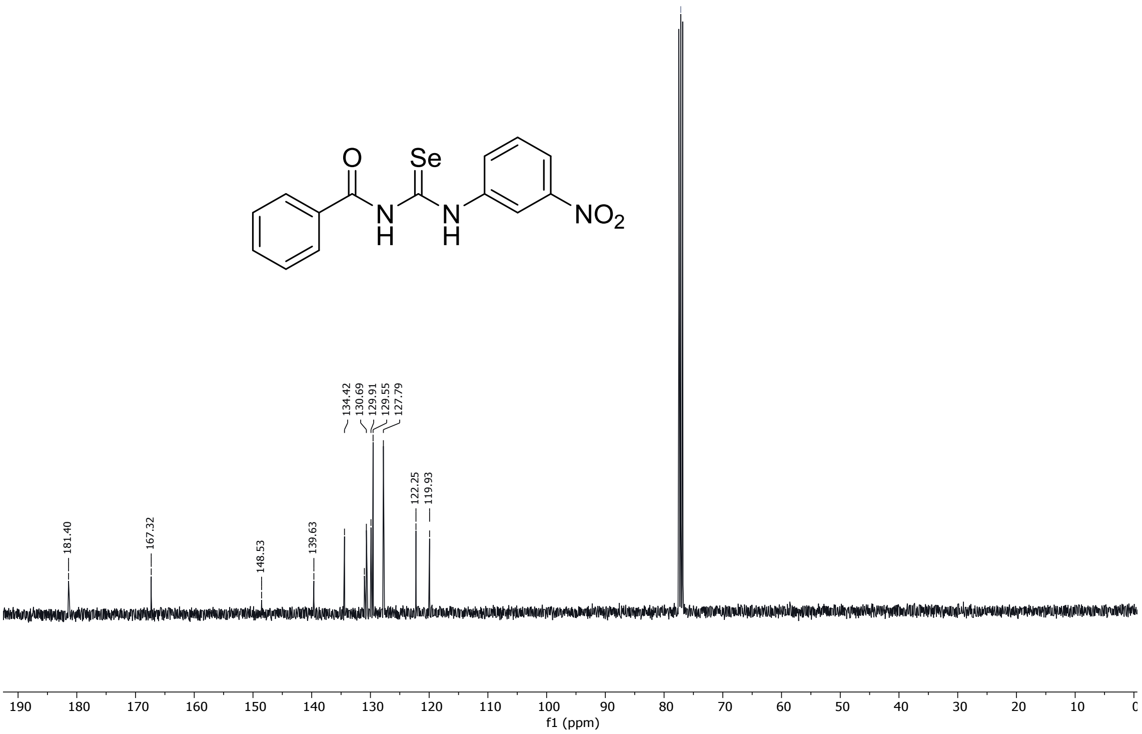

Figure S32: ^{13}C NMR (100 MHz, $CDCl_3$) – compound **BSU11**

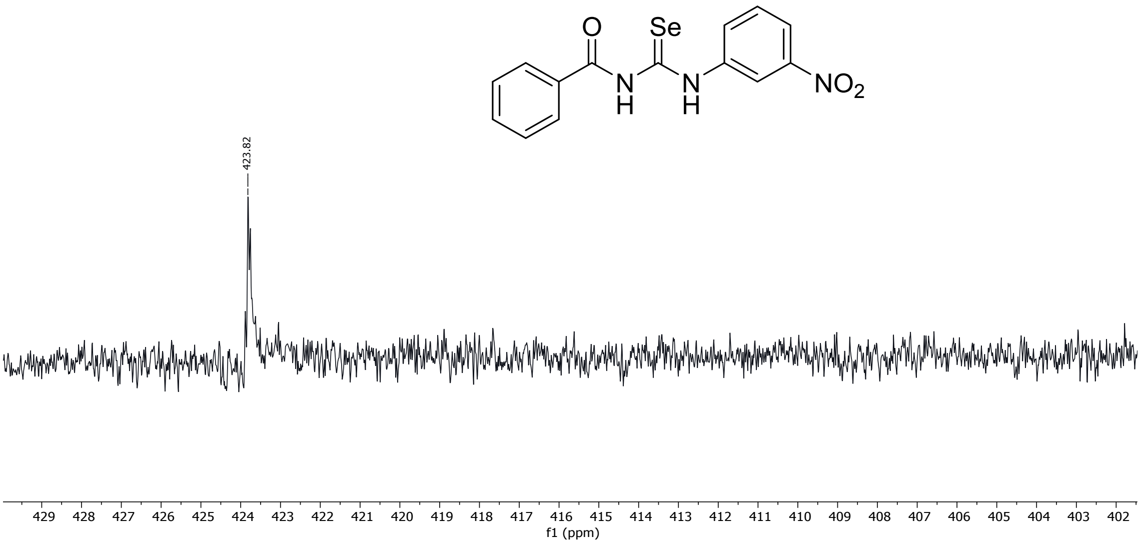

Figure S33: ^{77}Se NMR (51.5 MHz, $CDCl_3$) – compound **BSU11**

FTIR-ATR spectra of compounds BSU 1-11

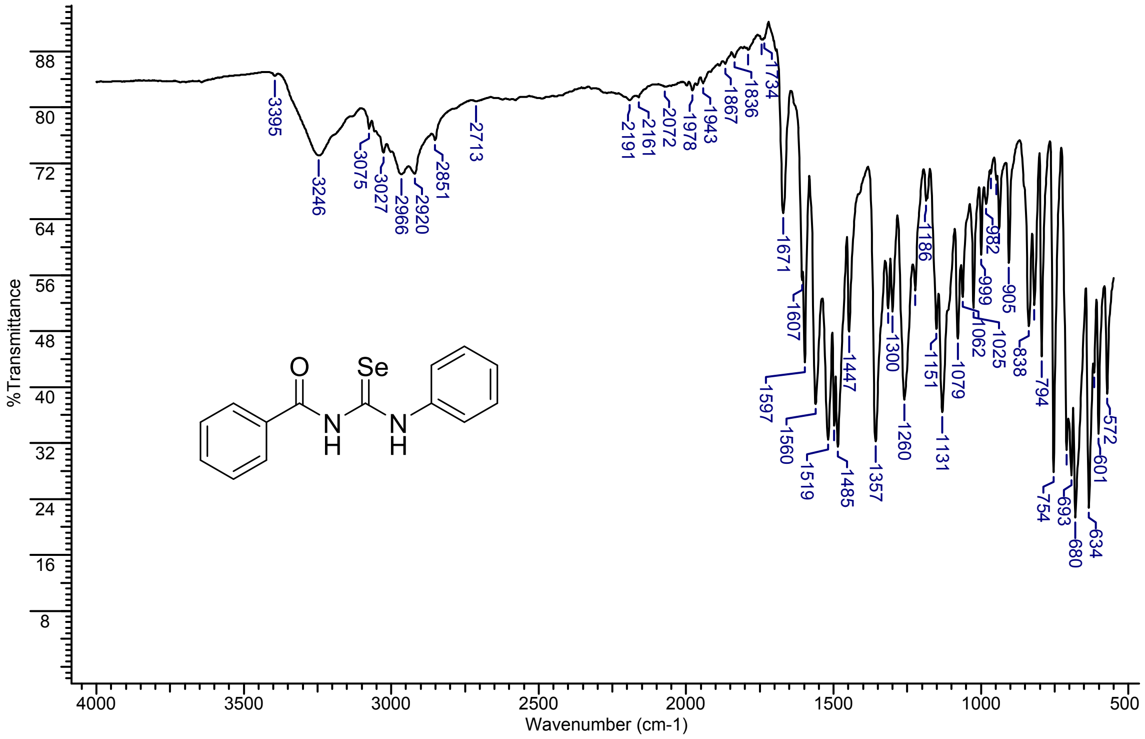

Figure S34: FTIR – ATR spectra of compound **BSU1**

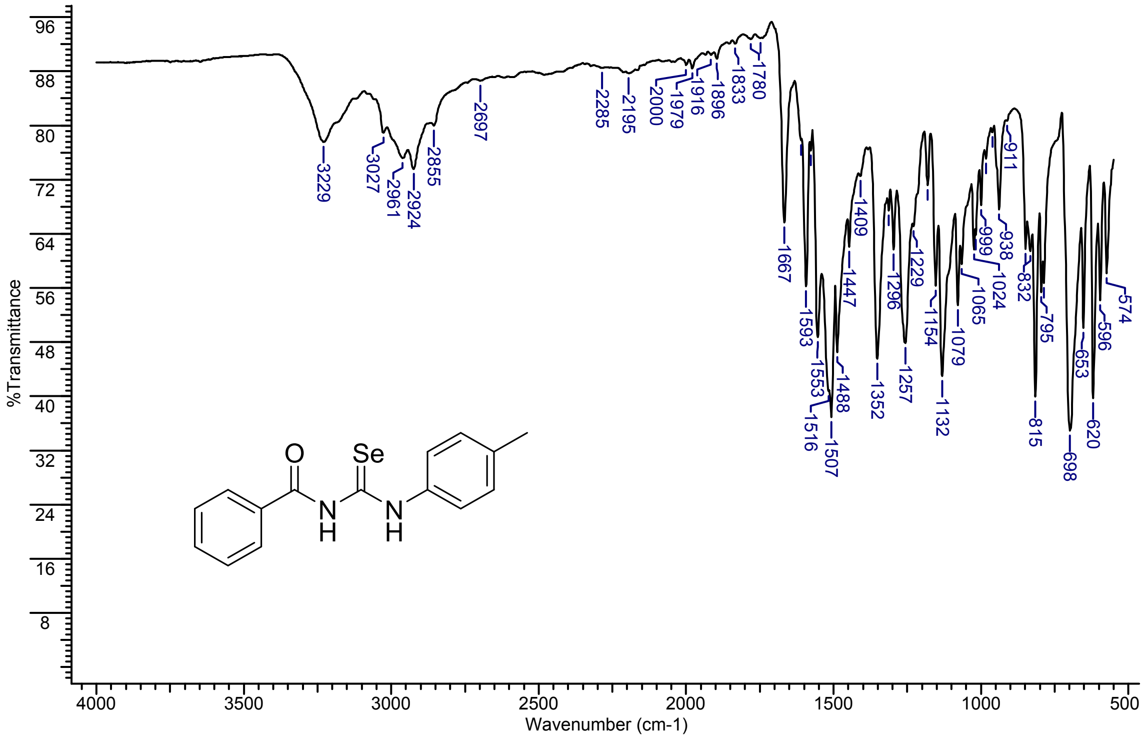

Figure S35: FTIR – ATR spectra of compound **BSU2**

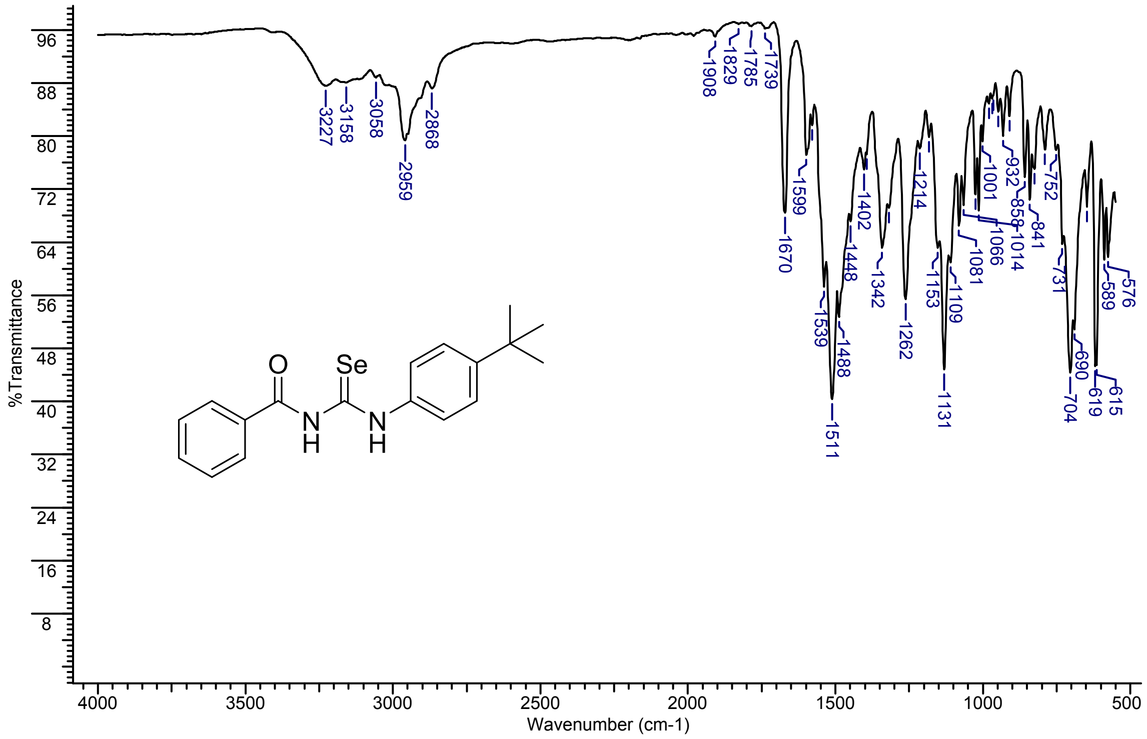

Figure S36: FTIR – ATR spectra of compound **BSU3**

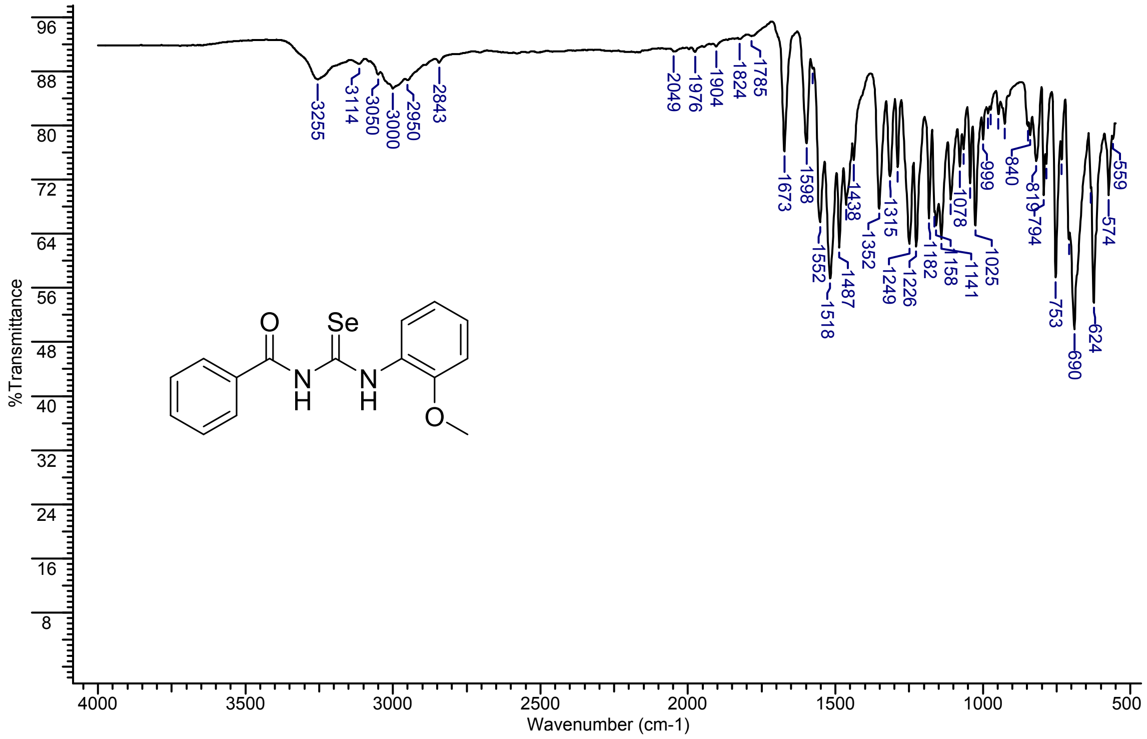

Figure S37: FTIR – ATR spectra of compound **BSU4**

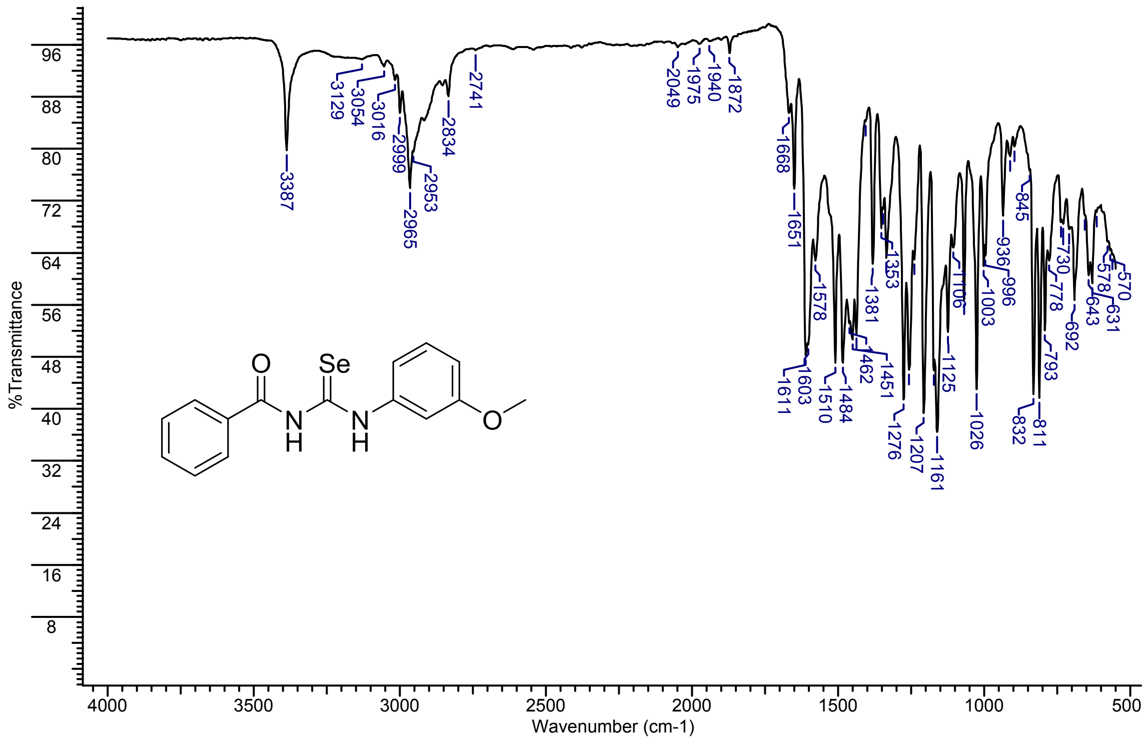

Figure S38: FTIR – ATR spectra of compound **BSU5**

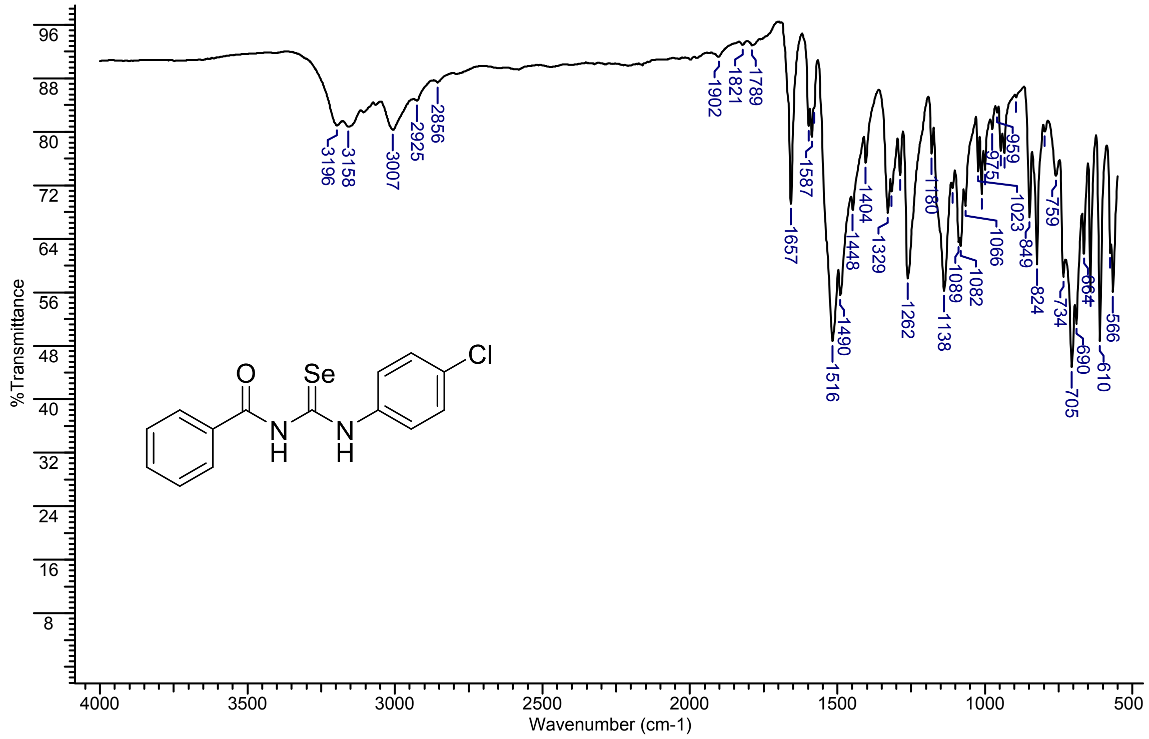

Figure S39: FTIR – ATR spectra of compound **BSU6**

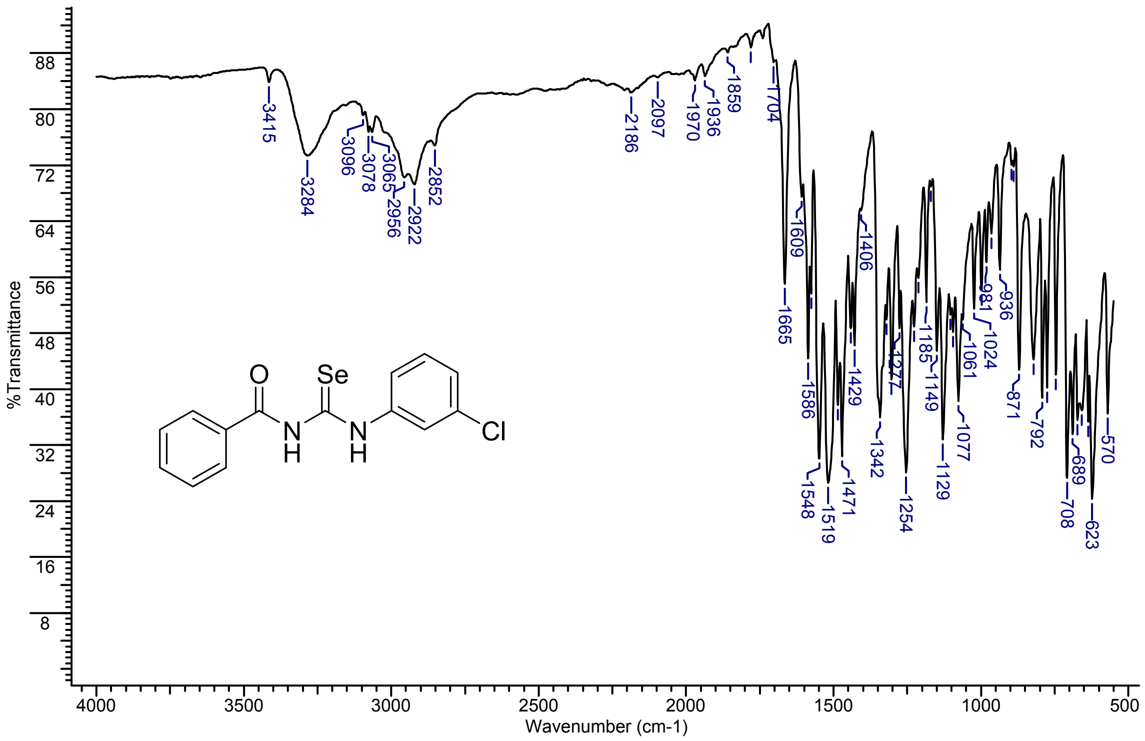

Figure S40: FTIR – ATR spectra of compound **BSU7**

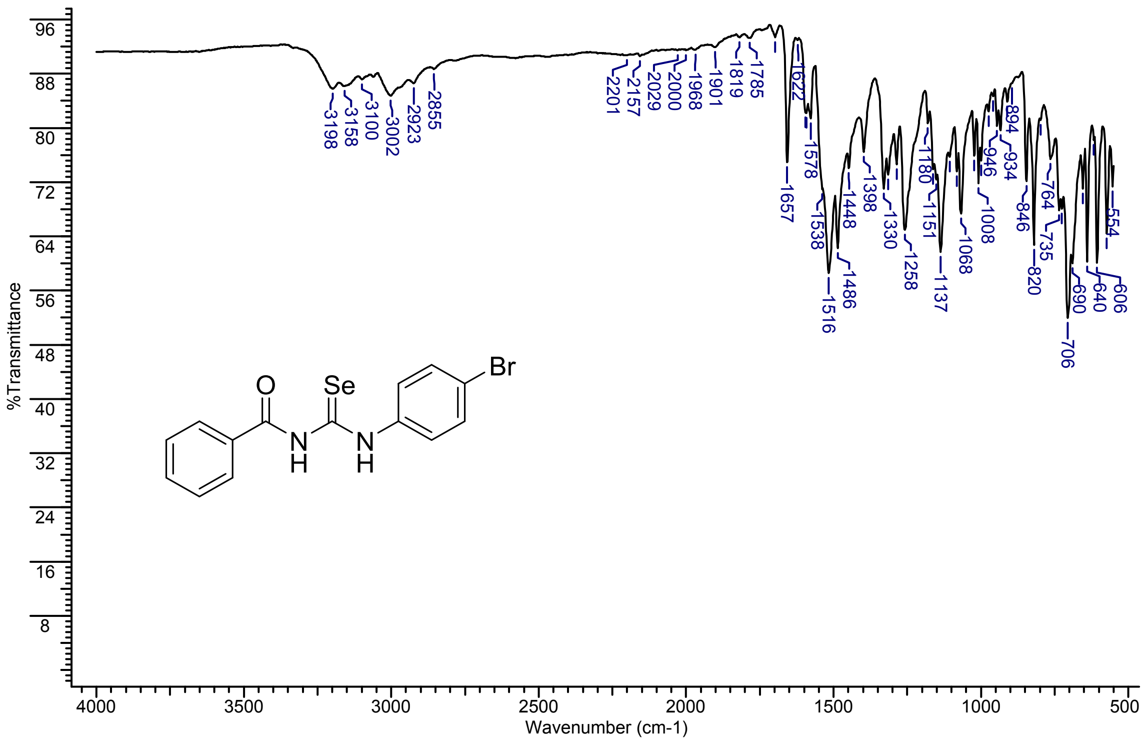

Figure S41: FTIR – ATR spectra of compound **BSU8**

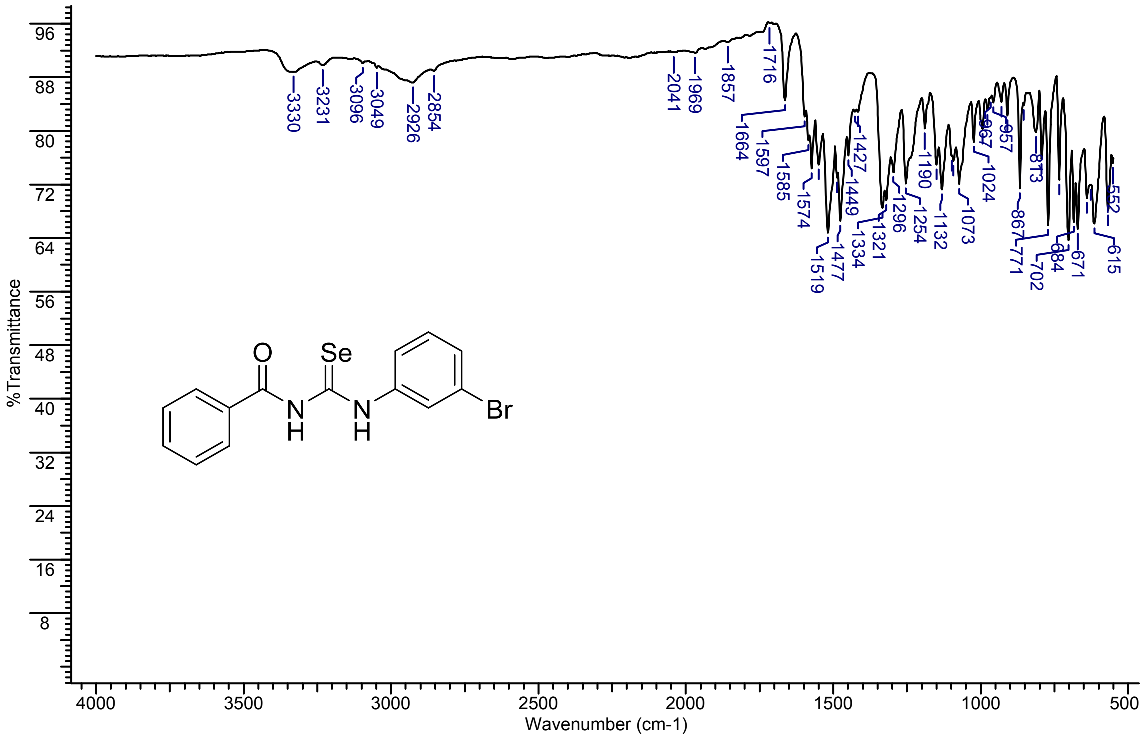

Figure S42: FTIR – ATR spectra of compound **BSU9**

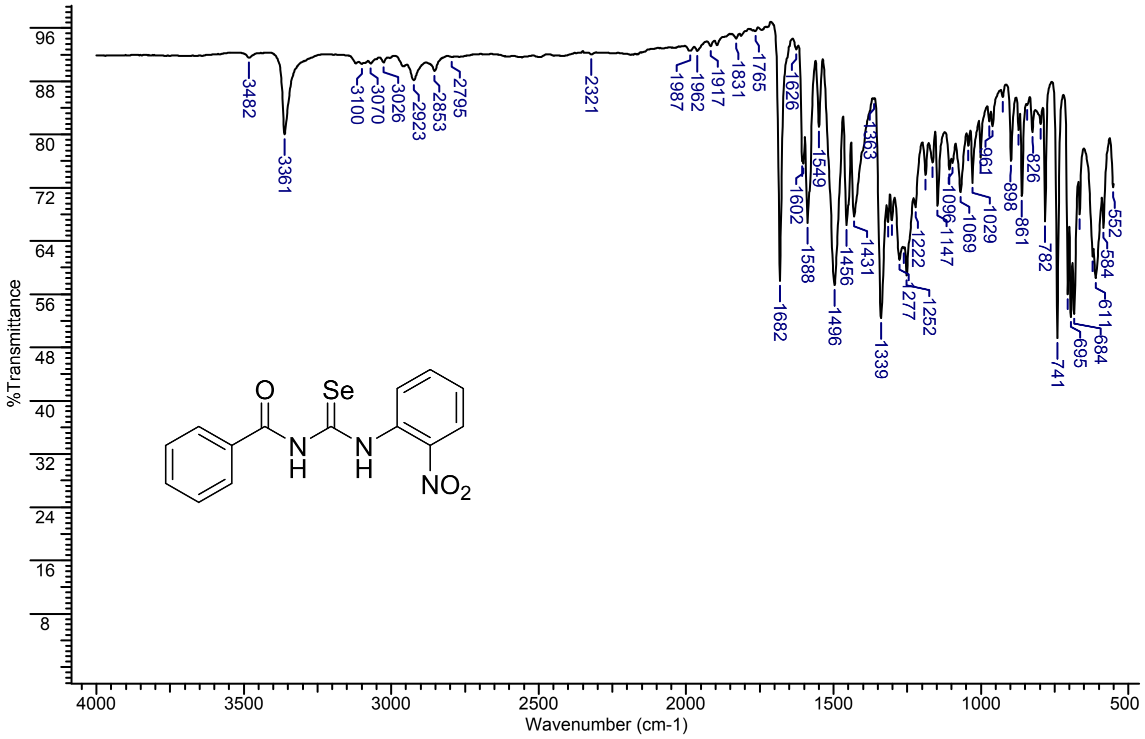

Figure S43: FTIR – ATR spectra of compound **BSU10**

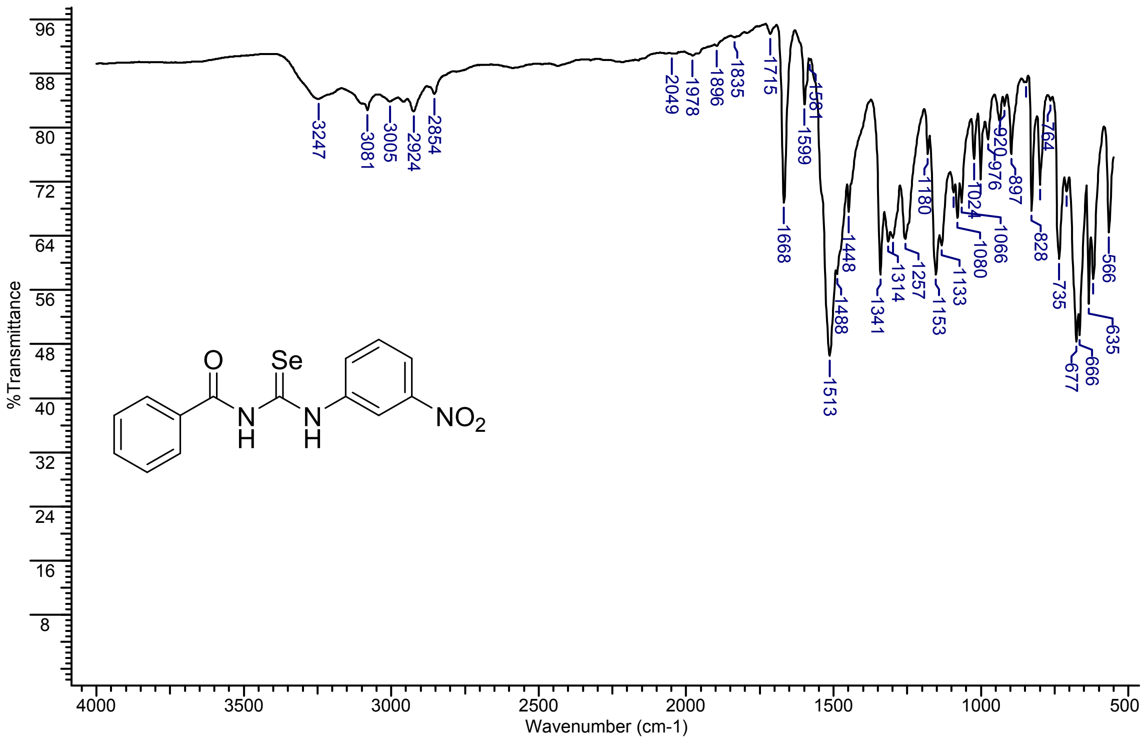

Figure S44: FTIR – ATR spectra of compound **BSU11**
